# Dual-frequency fiber-array photoacoustic computed tomography for high-resolution deep brain imaging

**DOI:** 10.1038/s41377-026-02324-3

**Published:** 2026-06-01

**Authors:** Zitao Chen, Yuhan Wu, Hexiang Xu, Lanling Liang, Jun Ma, Yi Zhang, Bai-Ou Guan

**Affiliations:** 1 Guangdong Provincial Key Laboratory of Optical Fiber Sensing and Communications, Institute of Photonics Technology, Jinan University, Guangzhou, China; 2 College of Physics & Optoelectronic Engineering, Jinan University, Guangzhou, China; 3 College of Life Science and Technology, Jinan University, Guangzhou, China

**Keywords:** Imaging and sensing, Photoacoustics

## Abstract

Photoacoustic tomography as an optical-ultrasound hybrid imaging modality provides rich optical contrast over the extended penetration depth of biological tissues, enabling multiscale multicontrast structural and functional imaging. However, inherent limitations in the state-of-the-art piezoelectric transducer arrays of the photoacoustic tomography, including size-dependent sensitivity, narrow bandwidth, and high material rigidity, compromise the resolution, penetration depth, and functional assessment precision. Here, an arc-shaped fiber ultrasound transducer array with a sheet-like ultrasound focus is demonstrated for photoacoustic computed tomography. At the ultrasound focus, a low detection limit of ~ 5.2 Pa and a dual-frequency response spanning several octaves are achieved. Whole mouse brain imaging with a depth up to ~ 1.2 cm and a spatial resolution of ~ 70 μm in the cerebral cortex region is showcased. The blood oxygen saturation within the entire mouse brain and the brain tumors is visualized, and the assessment precision is improved by leveraging the dual-frequency response of the transducer array. The centimeter-scale imaging depth, fine resolution of the cerebral vessels, and improved precision in the blood oxygenation evaluation make the fiber-array photoacoustic tomography a competitive candidate to the sought-after magnetic resonance imaging and ultrasound localization microscopy for brain functionality study and disease diagnosis.

## Abstract (web summary)

We develop an arc-shaped dual-frequency fiber ultrasound transducer array with sheet-like focus, enabling whole mouse brain imaging up to 1.2 cm depth and precise blood oxygen saturation mapping in glioblastoma.

## Graphical abstract

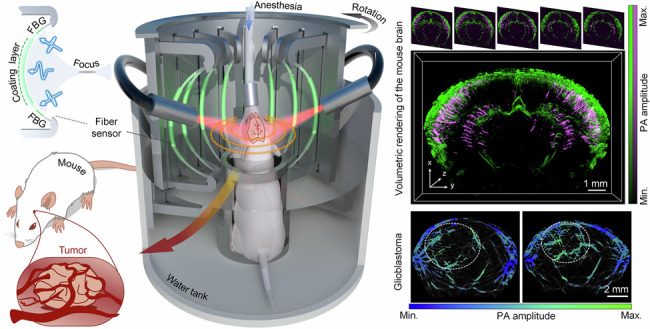

## Introduction

Biomedical imaging technologies with the capability to visualize structure, composition, and dynamic change of the target region in biological samples or patients become indispensable tools for fundamental research and clinical diagnosis^1–3^. For example, magnetic resonance imaging (MRI) and X-ray-based computed tomography (CT) achieve full penetration of the human body with a millimeter to submillimeter spatial resolution, providing insightful information for clinical disease screening and diagnosis. A major concern with MRI currently is its high cost and restriction for patients with metal implants, and a key limitation of CT is the risk of radiation exposure, hindering regular examinations for effective treatment and postoperative monitoring. Optical and ultrasound imaging modalities such as optical microscopy and ultrasound sonography are arguably biosafe, while the former suffers from shallow penetration depth due to the optical scattering and the latter lacks imaging contrast for the soft tissues. In the last decade, ultrasound localization microscopy capable of deep super-resolution vascular imaging by capturing the transient signal decorrelation of inert gas microbubbles has undergone fast development. The technique is powerful for resolving the microvasculature up to a centimeter-scale depth. However, it requires the injection of a microbubble contrast agent accompanied by the time-consuming localization reconstruction. It also lacks the capability for extravascular imaging and visualizing the total hemoglobin concentration (HbT) and the oxygen saturation (sO_2_), which is vital to the brain function and disease study^4–7^.

Photoacoustic tomography (PAT), also termed optoacoustic tomography, emerges as a noninvasive and hybrid imaging modality that integrates the high optical contrast of pure optical imaging with the large penetration depth of ultrasound imaging, representing a new trend for multiscale imaging from biological cells to human organs^8–10^. Through illumination of the entire region of interest (ROI) with diffused light and multi-point detection of the ultrasound emitted from the biological tissues, the photoacoustic computed tomography (PACT) in the configuration of the linear, ring, or spherical piezoelectric transducer arrays can achieve large-area and deep-penetration imaging after reconstructing the image with the time-of-flight information of the ultrasound. The technology has demonstrated great potential in various clinical scenarios, including the screening and subtyping of tumors, assessment of disease activity in inflammatory diseases, diagnosis of cardiovascular diseases, quantification of severity in dermatological conditions, and functional evaluation of peripheral arterial disease^11–14^. Typical examples, including the multispectral optoacoustic tomography (MSOT) system from iThera Medical and the optoacoustic plus ultrasound imaging system from Seno Medical, have devoted significantly to the global clinical translation^11,12^. Nevertheless, the image quality for the current PACT system still falls short of expectation as restricted by the piezoelectric transducers in aspects of their size-dependent sensitivity, narrow resonance bandwidth, and material rigidity. Firstly, the intrinsic tradeoff between the size and sensitivity causes the bulky size of the piezoelectric elements to obtain sufficient sensitivity, which reduces the acceptance angle and thus degrades the in-plane resolution. Secondly, the strong resonance of the piezoelectric elements leads to a relatively narrow frequency response range even after proper mechanical damping. The narrow frequency band not only leads to the information loss due to the heterogeneous biological tissues that generate photoacoustic (PA) signals in a wide frequency distribution^15^, but also causes compromise between the spatial resolution and the penetration depth^16–19^. To achieve fine spatial resolution and large penetration depth, the transducers or transducer arrays with typical frequencies ranging from sub-megahertz to several megahertz are combined with those at several tens of megahertz to capture both the low- and high-frequency signals, which account for large tissue penetration and high spatial resolution, respectively. However, the signal alignment and the image co-registration remain a challenging and non-trivial task^20,21^. Finally, the common piezoelectric ultrasound transducers are rigid, and their spatial responses are fixed once manufactured. Without the flexibility to reconfigure the spatial response, including the field of view (FOV) and focal depth, individual imaging systems are required to adapt to specific imaging scenarios such as the mouse brain imaging or the human breast imaging. Recently developed capacitive micro-machined ultrasound transducers (CMUTs), piezoelectric micro-machined ultrasound transducers (PMUTs)^22–27^, and on-chip optical resonators^28–31^ exhibit increased frequency bandwidth and reduced footprint, but the electrical signal interference between the elements, the insufficient sensitivity, or the sophisticated signal readout scheme hinder in vivo high-resolution deep-tissue imaging of biological tissues, especially for the mouse brain with severe ultrasound attenuation induced by the high-density tissue and the rigid skull bone.

Here, by leveraging the freedom in the lateral and the axial dimensions of the optical fiber transducers to tailor their ultrasound response, an all-optical PACT system based on an arc-shaped fiber ultrasound transducer (FUT) array with sheet-like ultrasound focus, high sensitivity, and dual-frequency band is developed. The FUT array-based PACT system shows improved spatial resolution, penetration depth, and quantitative precision for anatomic and functional imaging, aiming to overcome the inherent limitations in the state-of-the-art piezoelectric transducers. At first, by rational design of the polymer coating, the mechanical coupling between the bare silica fiber with a diameter of 125 μm and the polymer coating redistributes the energy of the oscillation excited by the impinging ultrasound waves from intrinsic transverse-resonant modes in the high-frequency range (20–30 MHz)^32^ to the low-frequency (2–3 MHz) range. This process enables dual-frequency ultrasound detection with the central frequency spanning over several octaves, which avoids the signal alignment and image co-registration if multiple transducers with different working frequencies are used. Then, the fiber of each array element is bent into a curvature radius of 4 cm to form ultrasound focusing by leveraging the high flexibility of the optical fiber along its axial direction. The lens-less ultrasound focus with a sheet-like pattern endows the system with a low-pressure detection limit down to ~ 5.2 Pa. It also brings out superior sectioning capability with a slice thickness of ~ 400 µm in the elevational direction, which significantly reduces the interference from out-of-plane signals. An arc-shaped PACT system with 150° angular coverage is then constructed with eight FUTs. The dual-frequency FUT array-based system demonstrates mouse brain imaging with the penetration depth up to ~ 1.2 cm and a high spatial resolution of ~ 70 μm in the cerebral cortex region. In addition, the dual-frequency response of the FUT array can capture not only the high-frequency PA signals responsible for the sharp vessel boundaries but also the low-frequency ones associated with the uniform absorption of the blood flowing inside the vessels. This feature improves the precision in quantitative assessment of the sO_2_, which is verified by both the phantom and in vivo experiments. The system is further applied for the visualization of the hemodynamics in the mouse brain subjected to oxygen (O_2_) challenge, as well as the mapping of the sO_2_ inside the mouse primary glioblastoma (GBM).

## Results

### Dual-frequency response

**Fig. 1:**
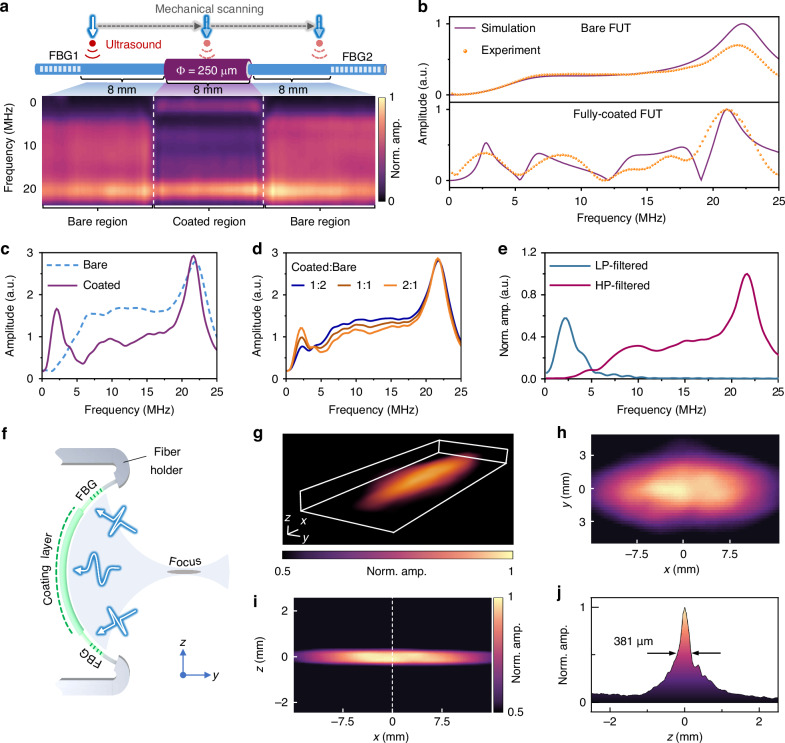
Ultrasound response of dual-frequency FUT. **a** Frequency response characterized by scanning the point-like PA source along the axial direction of a partially-coated FUT. The effective length of the cavity is ~ 24 mm, including the coating part with a diameter of 250 μm and a length of ~ 8 mm and the two-side bare parts with a diameter of 125 μm and lengths of 8 mm. **b** Simulated and experimental frequency responses of the bare and fully-coated FUTs to plane ultrasound waves. **c** Frequency responses to spherical ultrasound waves for the coated and bare FUTs obtained by integrating over the coated and bare regions in (**a**), respectively. **d** Frequency responses obtained by weighting average of the curves in (**c**) with different length ratios of the coated region to the bare region. **e** HP- and LP-filtered frequency spectra of the dual-frequency FUT, verifying the independent detection of high-frequency ( ~ 20 MHz) and low-frequency ( ~ 2.5 MHz) components. **f** Schematic of the focused FUT: the fiber is mechanically bent to form a sheet-like ultrasound focus. **g** Volumetric rendering of the measured spatial ultrasound response. **h**, **i** 2-D spatial ultrasound response in the *x-y* and *y-z* planes. **j** Profile along the dashed line in (**i**). Norm. amp.: normalized amplitude

The ultrasound response of a partially-coated FUT is characterized along the fiber axial direction as shown in Fig. 1a. For the FUT, the first fiber Bragg grating (FBG), denoted as FBG1, has a length of 12 mm, and the second FBG, denoted as FBG2, has a length of 20 mm. The region between the FBGs has a length of 24 mm, where the bare Er/Yb co-doped fiber has a diameter of 125 µm and the polymer coating part has a diameter of 250 µm. By scanning a PA effect-based ultrasound emitter, the ultrasound response is recorded step by step (details in **Methods**). During the scanning, the fiber is slightly stretched to keep the FUT straight, and the ultrasound emitter is aligned perpendicular to the straight FUT and irradiated by 532 nm laser pulses. The corresponding frequency spectrum is obtained after the Fourier transform of the record time-domain PA signal. To understand the difference between the ultrasound responses of FUTs without and with the polymer coating, the finite element method (Supplementary Note 1) is employed to simulate their frequency responses to plane ultrasound waves with frequency in the range of 0–25 MHz, as shown in Fig. 1b. For the bare fiber, the frequency peak at the 22 MHz corresponds to the eigen vibration mode T_21_, where the subscripts denote the azimuthal and radial order numbers of the in-plane vibration modes of the fiber. For the coated fiber, the polymer coating with a much lower Young’s modulus than that of the silica behaves like a mechanical oscillator with a low spring constant. The mechanical coupling between the silica fiber and the polymer coating results in multiple resonant peaks with the frequency ranging from 2 to 25 MHz. The simulated results are in agreement with the experimentally characterized frequency responses of the bare and fully-coated fiber to a plane ultrasound wave generated by laser-illuminated gold thin film (details in **Methods**). As the FUT works as a line-shaped detector, the actual output is the integration of the ultrasound response over the sensitive region for the partially-coated FUT. The accumulated frequency responses along the coated and bare regions of the FUT in Fig. 1a are obtained after integration and shown in Fig. 1c, verifying the increased low-frequency component by the coating. The frequency responses for the FUTs with different ratios of the coated and bare fiber length are further investigated through a weighted average of the two curves in Fig. 1c. As shown in Fig. 1d, the amplitude increases at the low frequency near 2.5 MHz and reduces at the high frequency in the range of 5-20 MHz as the coating length becomes longer. This suggests that the frequency response of the FUT can be tailored by adjusting the length ratio of the polymer coating to the bare silica fiber (Supplementary Note 2). A coating length of 8 mm is finally chosen to balance the low- and high-frequency response and thus the penetration and spatial resolution for biological tissue imaging. To avoid the aliasing of the low- and high-frequency components during the imaging process, low-pass (LP) and high-pass (HP) filters with a cut-off frequency of 5 MHz are applied to the acquired PA signals, with a dual-band frequency response as shown in Fig. 1e.

To improve the signal-to-noise ratio (SNR) for deep tissue imaging, the FUT is bent into a curvature radius of 4 cm to form a sheet-like ultrasound focus as sketched in Fig. 1f. The ultrasound focus is characterized by scanning a point-like ultrasound emitter in three dimensions (3-D). The volumetric rendering of the FUT spatial ultrasound response is shown in Fig. 1g and the two-dimensional (2-D) profiles in the *x*-*y* and *x*-*z* planes are shown in Fig. 1h, i. As estimated from the profile along the dashed line in Fig. 1i, the ultrasound focus has a slice thickness of ~ 380 µm, corresponding to the elevational resolution of the fiber-array PACT system (Fig. 1j). This sheet-like focus can not only improve the FUT sensitivity at a large working distance of ~ 4 cm with a characterized pressure detection limit of ~ 5.2 Pa (Supplementary Note 3) but also reduce the interferences from the out-of-plane signals for 2-D cross-sectional imaging. A leaf phantom is imaged by a curved FUT with the sheet-like focus and a straight FUT without focus (Supplementary Note 4). The significantly increased contrast of the image from the curved FUT emphasizes the importance of the focusing capability for improving the image performance. The imperfect symmetry of the curve in Fig. 1j may be attributed to the non-ideal symmetrical bending of the focused fiber transducer. The spatial responses of the FUTs at different bending curvatures are also characterized (Supplementary Note 5). In general, a larger curvature radius corresponds to a longer focal length and weaker focusing, and thus a larger FOV and reduced response amplitude.

### In vivo photoacoustic computed tomography

**Fig. 2:**
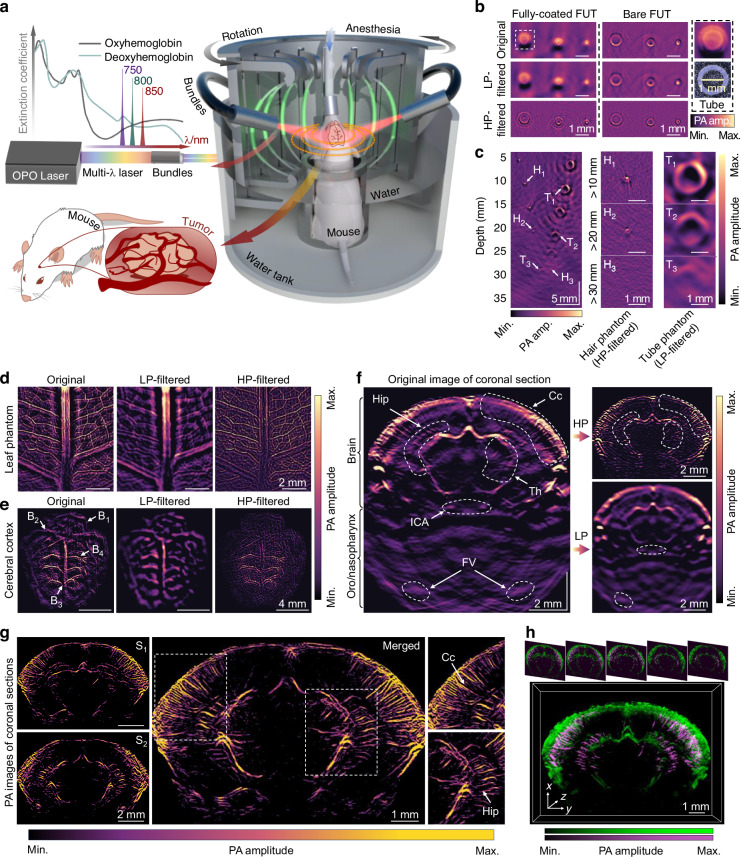
Schematic of the dual-frequency FUT array-based multispectral PACT system and imaging results. **a** Schematic of the PACT system. **b** PA images of solution-filled plastic tubes acquired by the fully-coated and bare FUTs. The reconstructed image of the tube from the original PA image acquired by the fully-coated fiber is enlarged to compare with the tube photograph. **c** PA imaging result of the hair and solution-filled tube hybrid phantom using the coated FUT. Intralipid is added to simulate the tissue scattering. The close-up images of the hairs (H_1_, H_2_, H_3_) and carbon nanoparticles-filled tubes (T_1_, T_2_, T_3_) at different depths are compared. **d** Original, LP- and HP-filtered PACT images of the leaf phantom. The cut-off frequencies are both 4 MHz. **e** Original, LP- and HP-filtered PACT images of the mouse cerebral cortical vasculature. The cut-off frequency is 2 MHz for the LP-filtered image and 4 MHz for the HP one. B_1_: sigmoid sinus, B_2_: transverse sinus, B_3_: superior sagittal sinus, B_4_: branch peripheral vessels. **f** PACT result of the coronal section of the whole mouse brain and oro/nasopharynx. Filtered results are shown as well, the LP cut-off frequency is 3 MHz and the HP one is 5 MHz. Cc: cerebral cortex, FV: facial vessel, Hip: hippocampus, ICA: internal carotid artery, Th: thalamus. **g** Coronal sections of the whole mouse brain. Two separate coronal sections of the mouse brain with 0.4 mm from the anterior to posterior as well as two close-up images for the areas within the dashed boxes are given. **h** Volumetric rendering of the mouse brain by stacking five successive coronal-section images

Figure 2a shows the all-optical PACT system based on the arc-shaped FUT array. The FUT array consists of eight optical fiber laser cavities, which are curved to form ultrasound focusing at the geometrical center plane. Each fiber laser consists of a piece of Er/Yb co-doped fiber sandwiched with two highly reflective FBGs. As aforementioned, the fiber polymer coating with a length of ~ 8 mm is retained to achieve efficient dual-frequency ultrasound detection. An optical parametric oscillation (OPO) laser with multiple output wavelengths is used as the excitation source for both the anatomic and functional imaging. To verify the importance of the dual-frequency ultrasound response, tubes filled with the carbon-nanoparticle solution are imaged by two FUTs with and without the coating in a circular-scanning configuration. The reconstructed images based on the back-projection (BP) algorithm from the data acquired by the two FUTs are compared in Fig. 2b. The coated FUT well recovers both the tube wall and the inner solution, whereas the bare FUT only captures the tube wall due to the loss of the low-frequency component of the PA signal, accounting for the homogeneous solution inside the tube. This difference can be more clearly observed from the images reconstructed using the PA signals HP- and LP-filtered with a cut-off frequency of 3 MHz. As the frequency of the PA signals scales with the sample size, fine-tuning of the cut-off frequency is performed to optimize the image quality for samples with different feature sizes. Due to the limited bandwidth of the conventional piezoelectric transducers and the increased ultrasound attenuation for higher frequencies in biological tissues, the previous PACT systems commonly employ pre-designed transducer arrays, either working at a high frequency for fine shallow tissue imaging or at a low working frequency for coarse deep tissue imaging. In comparison, the dual-frequency FUT supports the retrieval of both the high- and low-frequency PA signals, and thereby a single FUT-based PACT system can fulfill the need for different scenarios. As shown in Fig. 2c, both the hair with a small diameter of ~ 100 µm and the absorptive solution-filled tube with a diameter of 2 mm that are embedded into a scattering medium made of intralipid-mixed agar can be clearly observed. Compared to the hair phantom, the tube emitting a PA signal with a lower frequency is visible at an increased depth up to 3 cm. During the test, the laser pulse energy is adjusted to compensate for the difference in the optical absorption coefficient between the hair and the solution-filled tube.

Figure 2d, e illustrate the images of the leaf phantom and the mouse cerebral cortical vasculature, respectively. The bipolar images reconstructed by the BP algorithm are defined as the original PA images, which are subsequently decomposed into LP- and HP-filtered images. The LP-filtered image captures the primary vein of the leaf phantom but misses the fine structures, whereas the HP-filtered image offers the information of the tiny branches but only the edges of the primary vein. Similar results are found in the mouse cerebral cortex, where the LP-filtered image visualizes not only the superior sagittal sinus (SSS) and its cortical vascular branches but also the whole cerebral and hindbrain. Meanwhile, the HP-filtered signals complement the image with fine structures such as the small secondary vessels. The large FOV for the LP-filtered image results from the elongated ultrasound focus depth at low frequency (Supplementary Note 6 and 7).

Considering the ultrasound attenuation in brain tissues with a coefficient *α* = *a*·*f* ^*b*^ (dB cm^−1^·MHz), where the constants *a* = 0.8 and *b* = 1.35, the attenuation coefficient at 20 MHz is ~ 20 times larger than that at 2 MHz^33^. This exponential dependence of the attenuation on the ultrasound frequency severely limits the penetration depth of the high-frequency ultrasound, causing the loss of the deep-tissue information if adopting a high-frequency transducer array. As aforementioned, for the dual-frequency FUT, both the high- and low-frequency PA signals are acquired simultaneously and can be separated by simply applying a HP or LP filter. Figure 2f shows the original image of the mouse coronal section and the HP-and LP-filtered images as reconstructed from filtered PA signals. The cut-off frequencies of the 3rd order Butterworth filter are set to 5 MHz and 3 MHz for the HP and LP filters, respectively. Fine vessels with a diameter of ~ 70 µm in the shallow cortical region and a diameter of ~ 130 µm in the 4 mm-deep thalamus (Th) region can be resolved (Supplementary Note 8). Meanwhile, the large features across the whole mouse brain and even in the oro/nasopharyngeal region over a depth of 1.2 cm can also be visualized. Figure 2g further shows the detailed images of the mouse brain coronal sections at two different positions along the anteroposterior axis. Five coronal-section images acquired from different positions are merged to project the vasculature distributed in different cross sections of the mouse brain, including the cerebral cortex (Cc), the hippocampus (Hip), and the Th. The 3-D image of the mouse brain is also rendered by stacking the five coronal-section images as shown in Fig. 2h. These results confirm that the dual-frequency FUT array-based PACT system can penetrate throughout the whole mouse brain and simultaneously resolve the vasculature with various sizes in the cerebral cortex, which agrees with the simulated results (Supplementary Note 9).

### In vitro quantitative measurement of oxygen saturation

**Fig. 3:**
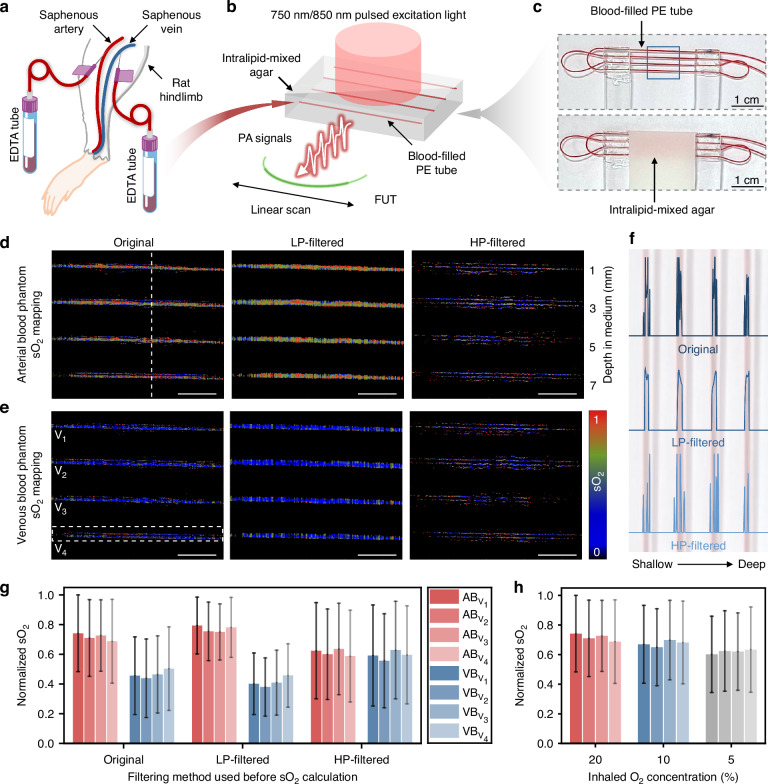
Visualization and statistical analysis of sO_2_ in the rat blood by the dual-frequency FUT. **a** Schematic of collecting arterial and venous blood samples from the rat saphenous artery and vein. EDTA: Ethylenediaminetetraacetic Acid. **b** Schematic of sO_2_ mapping of the blood-filled tube phantom embedded in intralipid-mixed agar by linear-scanning dual-frequency FUT. PE: polyethylene. **c** Photographs of the blood-filled tube before and after being embedded in the agar. sO_2_ maps of **d** arterial blood and **e** venous blood acquired with no filtering, LP- and HP-filtering. The blue dashed box in (**c**) denotes the sO_2_ mapping region. Scale bar: 2 mm. **f** sO_2_ profiles at the position along the white dashed line as denoted in (**d**) for different filtering conditions. **g** Statistical analysis of sO_2_ values between arterial and venous blood tubes at different depths. Non-zero values within phantom regions (schematically shown as the white dashed box in (**e**)) are analyzed, with all sO_2_ values normalized to the maximum of the four unfiltered arterial blood tubes. Bars and error bars represent normalized sO_2_ means and standard deviation (SD). **h** sO_2_ statistics from the blood samples under different inhaled O_2_ concentrations

The sO_2_ values estimated based on both the low- and high-frequency components of the PA signals are evaluated by measuring a blood-filled plastic tube that is filled in sequence with the arterial and venous blood freshly drawn from the rat inhaling O_2_ of different concentrations (Fig. 3a). As shown in Fig. 3b, a single FUT is linearly scanned across the tube phantom (Fig. 3c) with a 40 μm step size. Pulsed excitation light at 850 nm and 750 nm with a spot diameter of ~ 8 mm is delivered to the tube phantom through the optical fiber bundles. Prior to the sO_2_ analysis, the temporal PA signals are LP- or HP-filtered using filters with a cut-off frequency of 5 MHz. The three sigma standard deviations (3σ) of the background noise in the ROI for both the LP- and HP-filtered images are estimated as the threshold, and a mask excluding the pixels with the intensity below the threshold is applied to reduce the noise-related errors for the sO_2_ estimation. Representative low- and high-frequency sO_2_ maps of the blood-filled tubes of both arterial and venous blood under normal inhaled O_2_ levels for the rat are shown in Fig. 3d, e. The sO_2_ values at the corresponding locations (white dashed line in Fig. 3d) derived from different filtering processes are individually extracted and presented in Fig. 3f. While the high-frequency components resolve the sO_2_ information of the blood near the interfaces of the vessel walls, the low-frequency signals reflect the sO_2_ information of the whole blood inside the tubes.

Through further statistical analysis, the uncertainty of the sO_2_ values as acquired from the low-frequency sO_2_ maps for all four sections of the tubes at different depths as denoted by V_1_-V_4_ is lower than that from the high-frequency sO_2_ maps, as shown in Fig. 3g. In general, arterial blood is expected to exhibit hemoglobin sO_2_ levels substantially higher than those of venous blood. However, this fundamental characteristic is not observed from the sO_2_ values derived from HP-filtered PA signals due to the loss of the main blood sO_2_ information. In contrast, a significantly higher sO_2_ for the arterial blood than that for the venous blood is reflected by the sO_2_ mapping obtained from the LP-filtered PA signals, which coincides with the results acquired by a commercial blood gas analyzer (GEM Premier 3500, Werfen). As aforementioned, the low-frequency PA signals cover the whole cross-section of the blood flow in the tube, accounting for the true blood sO_2_ and reducing the sO_2_ fluctuation caused by the larger pixel-wise heterogeneity that exists for the high-frequency PA signals generated near the tube wall. Thus, the dual-frequency response of the FUT can enhance sO_2_ precision in biological tissues compared to the conventional single-band high-frequency transducer array-based PACT system.

In addition to the blood vessels as simulated by the tubes with a specific diameter of 300 μm, the dual-frequency response of the FUT can more accurately reproduce the sO_2_ levels of blood vessels at different sizes, from tens of micrometers to hundreds of micrometers, as verified by testing blood-filled tubes with various diameters (Supplementary Note 10). The influence of the blood flow velocity on the sO_2_ mapping results is also studied. Statistical analysis reveals that the changes in flow velocities from 0 to 20 mm s^−1^ have negligible influence on sO_2_, with a slight increase for a higher velocity (Supplementary Note 11). Considering the asynchronous acquisition of the PA images at different wavelengths, the possible influence on quantitative sO_2_ measurement for in vivo scenarios involving fast blood flow and sO_2_ dynamics will be investigated in the future after upgrading our system with a FUT array of more elements and a pulsed laser with faster wavelength switching. sO_2_ fluctuations of the arterial blood samples extracted from the rat subjected to varying inhaled O_2_ concentrations are further measured, and the results are shown in Fig. 3h. The increased precision of the sO_2_ measurement allows the monitoring of the dynamic sO_2_ changes in vivo. The detailed results for the comparative analysis of the sO_2_ maps in the mouse brain vasculature as obtained after the LP- and HP-filtered PA signals, are presented in Supplementary Note 12. Through the analysis similar to the blood-filled tube phantoms, the FUT capable of capturing both low- and high-frequency components of the PA signal can better restore the sO_2_ during the in vivo test for monitoring the physiological dynamics.

### Oxygen saturation monitoring in the mouse brain

**Fig. 4:**
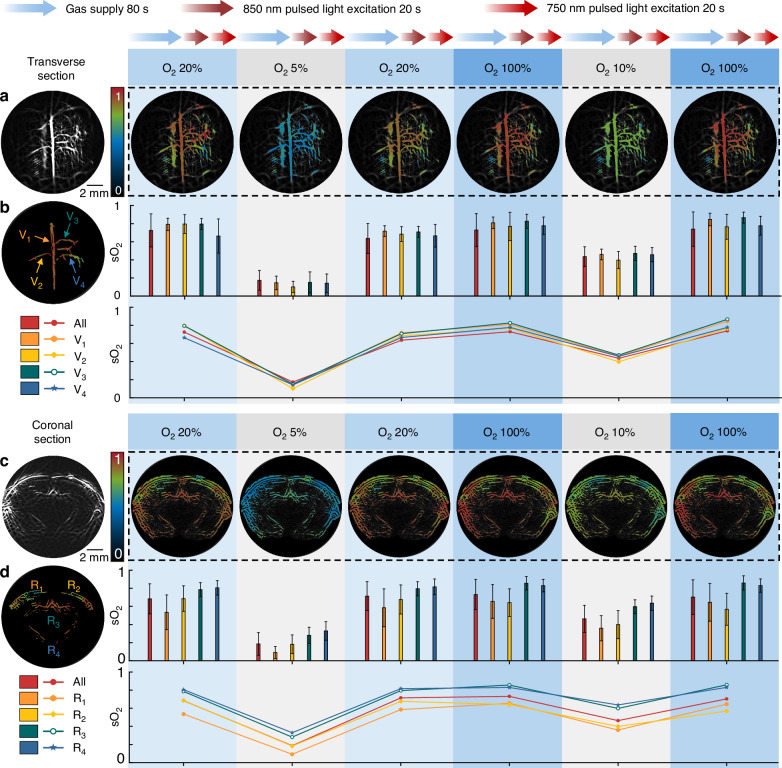
Dynamic sO_2_ change in the brain of the mouse under O_2_ challenge. **a** sO_2_ dynamics in the transverse section of the mouse brain during sequential exposure to four different O_2_ concentrations (5%, 10%, 20%, and 100%). **b** Quantitative analysis of sO_2_ for the total and the individual vessels (V_1_, V_2_, V_3_, and V_4_) in the transverse section as indicated by the arrows. sO_2_ values are presented as mean ± SD (above) and mean values (below). **c** sO_2_ distribution in the coronal section of the mouse brain under O_2_ challenge with the imaging protocol identical to that in (**a**). **d** Quantitative analysis of sO_2_ in the whole coronal section and four distinct anatomic regions (R_1_, R_2_, R_3_, and R_4_). sO_2_ values are presented as mean ± SD (above) and mean values (below)

The FUT array-based system is applied to sO_2_ monitoring in the brain of the mouse under the O_2_ challenge test. By leveraging the absorption difference of the oxygenated hemoglobin (HbO_2_) and the deoxygenated hemoglobin (Hb) at the wavelengths of 850 nm and 750 nm, the sO_2_ values can be obtained from the measured PA signals at these wavelengths after performing the linear spectral unmixing (see **Methods**)^34,35^. O_2_ challenges are performed by varying the inhaled O_2_ concentrations of the mouse and simultaneously imaging the transverse or coronal section of the mouse brain, as shown in Fig. 4. For each O_2_ concentration, a total 120 s O_2_ inhalation duration including ~ 80 s stabilization time and ~ 40 s imaging time is selected for our system to complete one round of sO_2_ measurement, which not only ensures that the cerebral sO_2_ of the animals reaches a steady state, but also avoids irreversible damage to the animals caused by prolonged hypoxia^17,36^.

During each cycle, the corresponding sO_2_ for both the transverse and coronal sections changes with the inhaled O_2_ concentrations of 20%, 5%, 20%, 100%, 10%, 100% (Fig. 4a, c) accordingly. In addition, four individual vessels from both the transverse and coronal cross sections, denoted as V_1_ to V_4_ and R_1_ to R_4_, respectively, are selected. Their sO_2_ changes follow a similar trend with the mean sO_2_ as estimated for all the vessels in the imaging region, as shown in Fig. 4b, d, verifying the capability of the system for monitoring the sO_2_ dynamics in hypoxic and hyperoxic conditions. Notably, obvious cerebral sO_2_ change can be observed for the change in the O_2_ level from 5% to 20% instead of 10% to 90% as in the previous study^17^. Moreover, the same trend for both the large superficial vessels such as the SSS (marked as V_1_ in Fig. 4b) and the deep-seated vessels in the cerebral peduncle regions at a depth of ~ 6 mm (marked as R_4_ in Fig. 4d) underscores the capability of the system for functional imaging of the biological tissue at diverse sizes and depths.

Currently, mechanical scanning is adopted to increase the spatial sampling rate and thus the image contrast, due to the limited element number of the array. The time for each frame is currently 20.8 s for a total of 208 scanning steps, considering the pulsed laser with a repetition rate of 10 Hz. To explore the potential of the system for continuous monitoring of the sO_2_, the originally acquired data containing 1664 A-lines is down-sampled for 10 times before the image reconstruction. Despite the degraded image quality due to the sparse sampling, the variation of the sO_2_ values can still well follow the changes of the applied O_2_ concentrations inhaled by the mouse (Supplementary Note 13). In the future, the acquisition time can be shortened and the mechanical scanning process can be avoided by increasing the array element number, which improves the temporal resolution and thus advances the system for real-time and high-fidelity sO_2_ mapping.

### Subcutaneous tumor and orthotopic glioblastoma screening

**Fig. 5:**
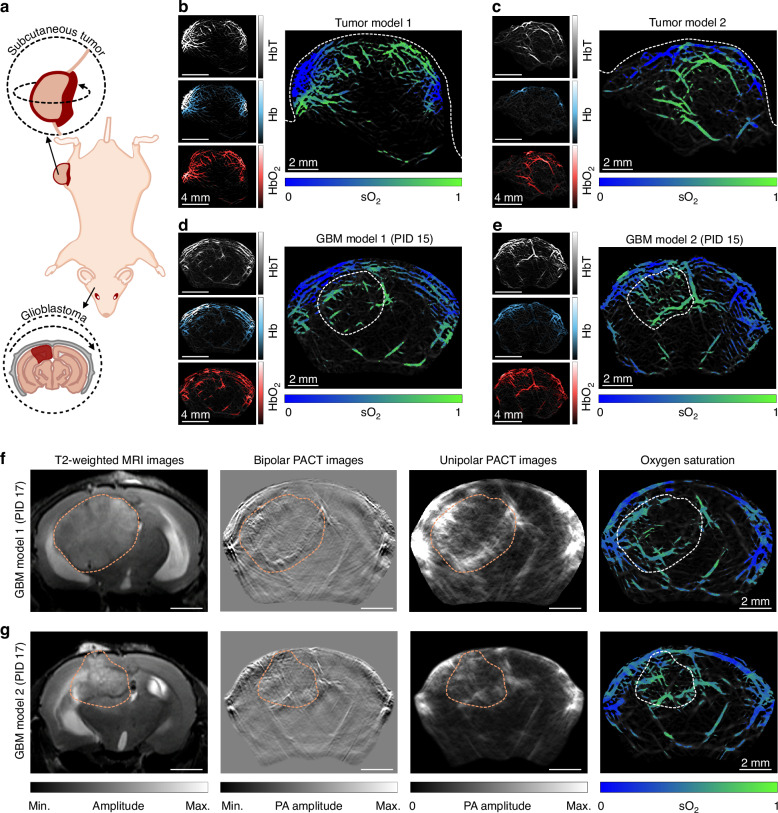
Multispectral imaging of neoplastic expansion and dysregulated sO_2_ in the mouse subcutaneous tumor and GBM. **a** Schematic of subcutaneous tumor and GBM in a mouse. The black dashed arrow indicates the scanning pathways of the FUT array during imaging. **b**, **c** sO_2_ imaging results of thigh subcutaneous tumors in two sets of mice, including HbO_2_, Hb, and HbT. **d**, **e** sO_2_ maps of the mouse brain coronal sections with GBMs. **f**, **g** MRI images, PACT anatomic images, and sO_2_ maps of the mouse brain coronal sections with GBMs. More coronal section images acquired by the MRI and PACT are presented in Supplementary Note 14. The dashed lines mark the margins of the subcutaneous tumors and the GBMs

To show the capability of the FUT array-based PACT system for screening tumors based on the abnormal changes in the anatomic structure and sO_2_, tumor-bearing mouse models are established by inoculation of GL261 cells. Figure 5a depicts the locations of two types of tumors in the mouse, i.e., the subcutaneous tumor and the orthotopic GBM. For the subcutaneous tumors, a palpable induration is observed at the injection site by post-inoculation day (PID) 9, indicating successful tumor implantation. Mapping of the sO_2_ in two subcutaneous tumors is performed at PID 15, with the results shown in Fig. 5b, c, together with the images of the HbT, Hb, and HbO_2_. The vasculature within the tumors is intricately branched and exhibits a relatively high-level sO_2_, which might result from the increased uptake of sufficient nutrients and O_2_ in the early stage for the proliferation and neoangiogenic differentiation of the tumor cells to form a neovascular system.

Identical imaging protocols are implemented on GBM-bearing mouse models at PID 15 with the results shown in Fig. 5d, e. Within the growth regions of the GBM as denoted by the white dashed lines, elevated sO_2_ is observed in the blood vessels. To verify the results, the coronal sections of two additional GBM-bearing mouse models are subsequently imaged by both the MRI and the PACT system (see Fig. 5f, g). The T2-weighted MRI images reveal heterogeneous tissues and deformed intracranial blood vessels due to the tumor compression, which can be well resolved in the PACT images as well. For better comparison, both the bipolar and unipolar PACT images are provided to compare with the MRI images, with the heterogeneous tissue contours outlined by the same dashed lines in light orange. The contours of the tumor regions in the MRI images are delineated by selecting the ROI encompassing the tumor, performing automatic contrast adjusting, and then manually selecting and connecting the points along the tumor boundary (Supplementary Note 14). The obtained tumor contours are then used as the segmentation reference and applied to the corresponding regions of the presented PA images to confirm the observed tumor vasculature as captured in Fig. 5f, g. The above results also coincide with the hematoxylin and eosin (H&E) staining of the brain slice (Supplementary Note 15) showing the presence of numerous mitotic cells within the tumor. More importantly, the vascular plexuses can be distinctly delineated in the sO_2_ maps of the tumor proliferative regions by the PACT system. The good correlation between the images of the hyper-oxygenated niches and neoplastic expansion from PACT and those from the MRI and histology reveals the ability of the multispectral PACT for effective tumor screening.

## Discussion

To address the inherent limitations of the state-of-the-art piezoelectric transducer arrays for high-performance PAT, a FUT array-based PACT system is developed based on optical detection of the ultrasound with high sensitivity, dual bandwidth, and reconfigurable sheet-like focus. Simultaneous detection of the ultrasound in the low-frequency region near 2.5 MHz and high-frequency region near 20 MHz can capture both the boundaries of blood vessels and the blood inside the vessels, which not only reduces the structural information loss but also improves the sO_2_ measurement precision for quantitative functional and molecular imaging. Both the fine vasculature in the mouse cerebral cortex and the general structural features across the whole mouse brain are visualized with high spatiotemporal coherence, avoiding the sophisticated alignment of low- and high-frequency images from two individual systems. By leveraging the flexibility of the FUT, lens-less sheet-like ultrasound focusing is realized by bending the fiber, which increases the sensitivity of the FUT array-based PACT system. This sheet-like ultrasound focus endows the system with a low detection limit down to ~ 5.2 Pa and superior sectioning capability with a slice thickness of ~ 400 µm in the elevational direction. An arc-shaped PACT array-based system with 150° angular coverage is constructed by eight dual-frequency FUTs and applied for whole-brain imaging of the mice with a depth up to ~ 1.2 cm and a high spatial resolution of ~ 70 μm for the cerebral cortex vessels.

Compared to the Bragg grating-based optical transducers^28,30,32,37–42^, the FUT realizes dual-band detection through controllable ultrasound coupling between the silica fiber and the surrounding polymer coating, providing the freedom to tailor its ultrasound response by tuning the coating length along the fiber axial direction. As the polymer coating is used to enhance the low frequency response, the bandwidth of ~ 21 MHz is larger than the previous fully bare or coated fiber FBG working in side-detection mode. Despite the bandwidth is still narrower than the Bragg gratings working in the tip-detection mode exhibiting a broad bandwidth over 100 MHz, the sheet-like ultrasound focus enables a lower detection limit of ~ 1 mPa Hz^−^^1/2^ and overcomes the sensitivity loss incurred by the ultrasound diffraction as confronted by the F-P cavity or Bragg grating-based optical transducers without focus^43^. The large FOV and long working distance also make the FUT well-suited for far-field imaging in animal studies and clinical applications. Performance comparison with the state-of-the-art Bragg grating-based optical transducers in terms of the bandwidth and sensitivity is given in Supplementary Note 16. In terms of the imaging performance, the current fiber PACT system captures far fewer vessels compared with the ultrafast ultrasound localization microscopy. However, as no microbubbles or dye droplets are injected and no time-consuming localization is performed, the imaging speed of the PACT system can potentially reach real time by increasing the number of the array elements. Moreover, the PACT system can provide the sO_2_ information, which is a critical parameter for studying neurovascular coupling in the brain^44^. In summary, the all-optical dual-frequency PACT system with high resolution, large penetration depth, and functional imaging capability provides a promising solution for high-performance anatomic, functional, and molecular imaging for biomedical research and clinical diagnosis.

## Materials and methods

### Preparation and characterization of the FUT

The FUT is fabricated by inscribing two FBGs at both sides of an Er/Yb-doped fiber with a 193 nm ultraviolet (UV) laser and a phase mask with a pitch of 1063.86 nm. Specifically, FBG1 has a length of 12 mm and a reflectivity of 98.4%, and FBG2 has a length of 20 mm and a reflectivity of 99.9%. The Er/Yb co-doped fiber and the FBGs form a 24 mm F-P cavity with the central wavelength of the reflection spectrum at 1542.8 nm. The cavity is pumped by a 980 nm continuous-wave laser with a power of 250 mW, which generates a stable laser output of ~ 16 mW. Due to the intrinsic fiber birefringence, the output laser includes two orthogonal polarization modes that beat with each other and generate radio frequency (RF) signals. Once ultrasound waves impinge on the fiber, the induced stress in the fiber shifts the beat frequency of the laser light. By demodulating the temporal variation of the beat frequency, the time-domain ultrasound signals can be retrieved.

For the FUT, only part of the polymer coating on the Er/Yb co-doped fiber between the two FBGs is stripped off. Due to the acoustic coupling between the bare silica fiber and the polymer coating, the frequency response is controlled by simply varying the polymer coating length. The frequency response along the fiber is obtained by scanning a PA ultrasound emitter made of a 100 μm diameter human hair. The 532 nm pulsed light with a spot size of 1 mm^2^ is used to irradiate the emitter. The FUT is kept straight by applying a slight stretching force and aligned perpendicular to the emitter at a vertical distance of ~ 1 mm. The scanning has 600 steps with a step size of 40 μm.

For the frequency response of the bare and fully-coated fiber to a plane ultrasound wave as compared with the simulated results in Fig. 1b, a glass slide with a thickness of 150 μm is coated with a 50 nm gold film, which is irradiated by a 532 nm pulsed light with an expanded spot of ~ 25 mm. To characterize the sheet-like spatial response, we mechanically scan a point-like ultrasound source made of a carbon-black-coated fiber tip with a step size of 400 μm. Ultrasound waves are generated by illuminating the fiber tip with 532 nm laser pulses. The experimental result of the spatial response for both single transducer and all sensor elements in the array system over an area of 3.5 cm × 3.5 cm is given in Supplementary Note 17. The FOV is defined as the full width at half maximum (FWHM) of the peak-to-peak envelope of the profiles along the *x* and *y* axes and is ~ 16.2 mm × 10.4 mm. The smaller FWHM along the *y* axis is caused by the 150° coverage angle of the ring array, not fully 180°.

### FUT array-based PACT system

Eight FUTs with a fiber coating length of 8 mm are used for the PACT system construction. The coating length is selected to balance the low- and high-frequency responses, thus the imaging depth and the spatial resolution. To achieve elevational focusing, FUTs are bent to an arc shape with a radius curvature of 40 mm under the assistance of the customized holders that fix the fibers on two sides. FUTs have a focus length equal to the radius curvature and are circumferentially arranged with an angular interval of 18.75° along the focus center. The FUT array holder has a radius curvature of ~ 40 mm and an opening angle of 150°. All the foci of the eight FUTs are aligned to be confocal by imaging a hair phantom profile and then fixed. The sensitivity, uniformity and stability of the system during the mechanical scanning are evaluated experimentally (Supplementary Note 3). The noise level for all elements actually experiences slight variation, which is found to induce a negligible influence on the imaging performance. To characterize the spatial resolution of the FUT array-based PACT system, 10 μm polystyrene dyed microspheres are utilized. Based on the acquired image of the microspheres, the characterized system axial and lateral resolutions are ~ 68 μm and ~ 74 μm, respectively, with the results given in Supplementary Note 18. The build-up of the FUT array-based PACT system is detailed in Supplementary Note 19. The FUT array is interrogated by an eight-channel demodulator that allows parallel interrogation of all the array elements. Pulsed light from the OPO laser is delivered to the imaging targets through the Y-shaped fiber bundles for the excitation of PA signals. Laser pulses of multiple wavelengths (532, 750, 800, 850, and 1064 nm) are employed with corresponding target surface energy densities of approximately 17, 19, 16.5, 14, and 50 mJ cm^−2^, respectively, for imaging experiments, including in vitro blood sO_2_ test, the leaf phantom and mouse brain imaging, and the tumor screening. All of the radiation influences adopted during the imaging processes are below the limits of the American National Standards Institute (ANSI). More specifically, 532 nm laser pulses are used for the leaf phantom and the mouse cerebral cortex imaging. 532 nm and 1064 nm pulses are employed for the tube cross-section imaging as shown in Fig. 2b, c, respectively. 850 nm pulses are used for the mouse brain coronal section. 750 and 850 nm pulses are used for the in vitro blood sO_2_ measurement and mouse O_2_ challenge test. 750, 800, and 850 nm pulses are used for the tumor screening. During anatomic imaging of phantoms and mouse brains, a single FUT with the sheet-like focus subjects to a circular scan of ~ 152.7°. A total of 1400 A-lines, each containing 3072 data points, is acquired with an imaging time of 140 s. For the sO_2_ mapping of the blood-filled tubes, a single FUT is linearly scanned in 400 steps with a step size of 40 µm. For the mouse brains and tumors, the FUT array is circularly scanned for ~ 18.72°, and the total coverage angle of these eight elements is ~ 150°. 208 A-lines are acquired by each element during a period of 20.8 s, corresponding to a total of 1664 A-lines for the eight-element array.

### Phantoms preparation

The Teflon tube phantom consists of three tubes with the inner diameters (IDs) of 0.4 mm, 0.7 mm, and 1 mm. The carbon-black solution after 200-fold dilution is injected into the tubes as absorptive material. Both ends of each tube are sealed by UV resin and embedded parallel inside a solid mixture of 3% agar and 1% intralipid emulsion. The intralipid emulsion is added to simulate the optical scattering in the tissue. The phantom is placed at the focal zone of the array and the 532 nm pulsed light is expanded to an illumination area of 1 cm × 2.5 cm.

The hair-tube hybrid phantom consists of human hairs with a diameter of ~ 0.1 mm and the Teflon tubes with an ID of ~ 2 mm. The tubes are filled with the carbon-black solution. The hairs and solution-filled tubes are immobilized in a cylinder-shaped solid mixture of 3% agar and 1% intralipid emulsion, giving an entire cylindrical phantom with a diameter of 6 cm. The interval between the tubes is ~ 5 mm, equal to that between the hairs. The phantom is positioned at the array center and illuminated by the 1064 nm pulsed light incident from the sides of the phantom. The leaf phantom is prepared by embedding a part of a leaf with both the main vein and branches into 3% agar.

For the blood-filled tube phantom, arterial and venous blood samples are collected from the saphenous artery and vein in Sprague-Dawley rat (8 weeks old, Charles River) hindlimbs using single-use evacuated tubes (EDTAK2, Hebei Kangweishi Medical Technology Co., Ltd.), with all samples utilized for sO_2_ measurements within 30 mins post-collection. Inhaled O_2_ concentrations for the mice are regulated through controlled blending of pure nitrogen (N_2_, purity ≥ 99.999%, Guangzhou Yigas Co., Ltd.) and air (20.95% O_2_ mixed with balanced N_2_, Dalian Special Gases Co., Ltd.) via the mass flow controller (CS-200, Sevenstar, Beijing Aurasky Electronics Co., Ltd.). Each gas mixture is administered for 80 s to achieve steady-state blood gas equilibrium, immediately followed by the blood sampling to capture the sO_2_ responses. Polyethylene (PE) tube (outer diameter: 400 μm; ID: 300 μm) is slightly stretched and coiled on a slotted acrylic plate, parallelly arranged with an interval of ~ 2 mm. The blood is infused into the tube through the tube ends. A mixture solution with 3% agar and 1% intralipid emulsion is poured into the slots of the acrylic plate to evenly wrap the PE tube and then solidified. The horizontally positioned PE tube is infused with blood for imaging. After the imaging process, the tube is flushed with saline to evacuate residual blood for the next test.

### In vivo mouse brain imaging

BALB/c mice (4–6 weeks old, Charles River) are used for in vivo imaging. The mouse is first anesthetized by a mixture of 1.5% isoflurane and the air at a flow rate of ~ 0.5 L min^−1^. The isoflurane is reduced to 0.75% during the imaging process to ensure the survival of the mouse. The hair is removed, and the skull is thinned to reduce the ultrasound attenuation. The thinned skull region ranges from 1 to 3 mm posterior to the bregma, with a width of 8 mm centered at the SSS. Two types of fixations for the mouse are employed during the mouse brain imaging process. For the cerebral cortical vascular imaging, the mouse is placed in a lab-customized holder with cephalic fixation, and the respirator mask is continuously ventilated with the anesthetic gas mixture. The mouse head is applied with ultrasound coupling gel and then covered by the ultrasound-permeable polymeric film. For the coronal cross-section imaging, the mouse body is fixed on a customized holder and immersed in a temperature-regulated water tank. Silicone rubber sealant is applied at the gap between the mouse head and the mask to prevent water infiltration. Once the imaging procedure is completed, the mouse is dried on a heating pad to recover. All procedures are carried out in accordance with the Institutional Animal Care and Use Committee at Jinan University.

During the O_2_ challenge, the O_2_ concentration of the mouse inhaled gas is regulated by varying the proportion of pure O_2_, pure N_2_, and the air via the mass flow controllers. For each O_2_ concentration, a period of 80 s is left to wait for the stabilization of sO_2_ in the mouse brain prior to the imaging procedure. Dual-wavelength PACT imaging at 850 nm and 750 nm is performed sequentially, and the imaging time for each O_2_ concentration is ~ 41.6 s.

### Mouse models for subcutaneous tumor and glioblastoma

BALB/c nude mice (8 weeks old, Charles River) are injected subcutaneously or stereotactically with GL261 cells to establish subcutaneous tumors and GBM models. For the subcutaneous tumor mouse model, 2 mice are inoculated with 1 × 10^6^ cells at the right hindlimb area to establish a single tumor. Once the average subcutaneous tumor diameter reaches 1 cm, the detection is performed. For the GBM mouse model, 2 mice are anesthetized with 3% isoflurane and stereotactically injected with 1 × 10^6^ GL261 cells in phosphate buffer saline (PBS) with a volume of 4 μL using a microinjection pump and an injection electrode, following the coordinates: 1.5 mm lateral, 2.0 mm posterior to bregma, 1.5 mm depth from the skull surface.

### Image reconstruction

The image is reconstructed based on the BP algorithm using MATLAB (R2024a, MathWorks). Before image reconstruction, the acquired PA signals are processed by the 3rd-order Butterworth filter to separate the low- and high-frequency components. The Hessian-based 2-D Frangi filter is applied to process the brain images for anatomic information. Volumetric rendering of the mouse brain by stacking the five coronal section images is performed via ParaView (v5.11.1, Kitware). By assuming a linear dependence of the PA signal intensity on the tissue absorption coefficient, the sO_2_ values are estimated based on linear spectral unmixing and given by,

1 $${{\rm{sO}}}_{2}(x,y)=\frac{{C}_{Hb{O}_{2}}(x,y)}{{C}_{Hb}(x,y)+{C}_{Hb{O}_{2}}(x,y)}\times 100 \%$$

2 $$\left[\begin{array}{l}{{\rm{C}}}_{Hb}(x,y)\\ {{\rm{C}}}_{Hb{O}_{2}}(x,y)\end{array}\right]={({\varepsilon }^{T}\varepsilon )}^{-1}{\varepsilon }^{T}P$$

In Eq. (2),

3 $$P=\left[\begin{array}{l}P({\lambda }_{1},x,y)\\ P({\lambda }_{2},x,y)\\ P({\lambda }_{3},x,y)\end{array}\right]$$

is the matrix of the image intensity for three wavelengths, where *λ*_*i*_ is the wavelength and (*x*, *y*) are the pixel coordinates.

4 $$\varepsilon =\left[\begin{array}{cc}{\varepsilon }_{Hb}({\lambda }_{1}) & {\varepsilon }_{Hb{O}_{2}}({\lambda }_{1})\\ {\varepsilon }_{Hb}({\lambda }_{2}) & {\varepsilon }_{Hb{O}_{2}}({\lambda }_{2})\\ {\varepsilon }_{Hb}({\lambda }_{3}) & {\varepsilon }_{Hb{O}_{2}}({\lambda }_{3})\end{array}\right]$$

is the extinction coefficient matrix, where *ε*_*Hb*_(*λ*_*i*_) and *ε*_*HbO2*_(*λ*_*i*_) are the extinction coefficients of the Hb and HbO_2_ at *λ*_*i*_. For the sO_2_ mapping that involves only 750 nm and 850 nm laser pulses for excitation, the terms for *λ*_*3*_ are omitted. The Hb and HbO_2_ concentrations *C*_*Hb*_(*x*,*y*) and *C*_*HbO2*_(*x*,*y*) can be calculated by Eq. (2).

## Supplementary information

Supplementary material

## Data Availability

All data needed to evaluate the conclusions are present in the paper and the Supplementary Information. Additional data is available from the authors upon request.

## References

[1] Weissleder, R. & Nahrendorf, M. Advancing biomedical imaging. *Proc. Natl. Acad. Sci. USA* **112**, 14424–14428 (2015). 26598657 10.1073/pnas.1508524112 PMC4664297

[2] Bischof, J. et al. Multimodal bioimaging across disciplines and scales: challenges, opportunities and breaking down barriers. *npj. Imaging* **2**, 5 (2024). 40603654 10.1038/s44303-024-00010-w PMC12118647

[3] Wallyn, J. et al. Biomedical imaging: principles, technologies, clinical aspects, contrast agents, limitations and future trends in nanomedicines. *Pharm. Res.* **36**, 78 (2019). 30945009 10.1007/s11095-019-2608-5

[4] Errico, C. et al. Ultrafast ultrasound localization microscopy for deep super-resolution vascular imaging. *Nature* **527**, 499–502 (2015). 26607546 10.1038/nature16066

[5] Renaudin, N. et al. Functional ultrasound localization microscopy reveals brain-wide neurovascular activity on a microscopic scale. *Nat. Methods* **19**, 1004–1012 (2022). 35927475 10.1038/s41592-022-01549-5 PMC9352591

[6] Demené, C. et al. Transcranial ultrafast ultrasound localization microscopy of brain vasculature in patients. *Nat. Biomed. Eng.* **5**, 219–228 (2021). 33723412 10.1038/s41551-021-00697-x PMC7610356

[7] Lin, B. Z. et al. Combined nanodrops imaging and ultrasound localization microscopy for detecting intracerebral hemorrhage. *Ultrasound Med. Biol.* **51**, 707–714 (2025). 39837748 10.1016/j.ultrasmedbio.2025.01.002 PMC12615983

[8] Wang, L. V. & Hu, S. Photoacoustic tomography: in vivo imaging from organelles to organs. *Science* **335**, 1458–1462 (2012). 22442475 10.1126/science.1216210 PMC3322413

[9] Wang, L. V. & Yao, J. J. A practical guide to photoacoustic tomography in the life sciences. *Nat. Methods* **13**, 627–638 (2016). 27467726 10.1038/nmeth.3925 PMC4980387

[10] Steinberg, I. et al. Photoacoustic clinical imaging. *Photoacoustics* **14**, 77–98 (2019). 31293884 10.1016/j.pacs.2019.05.001 PMC6595011

[11] Ntziachristos, V. Addressing unmet clinical need with optoacoustic imaging. *Nat. Rev. Bioeng.* **3**, 182–184 (2024).

[12] Knieling, F., Lee, S. & Ntziachristos, V. A primer on current status and future opportunities of clinical optoacoustic imaging. *npj Imaging* **3**, 4 (2025). 40603705 10.1038/s44303-024-00065-9 PMC12091691

[13] Lin, L. & Wang, L. V. The emerging role of photoacoustic imaging in clinical oncology. *Nat. Rev. Clin. Oncol.* **19**, 365–384 (2022). 35322236 10.1038/s41571-022-00615-3

[14] Park, J. et al. Clinical translation of photoacoustic imaging. *Nat. Rev. Bioeng.* **3**, 193–212 (2024).

[15] Aguirre, J. et al. Precision assessment of label-free psoriasis biomarkers with ultra-broadband optoacoustic mesoscopy. *Nat. Biomed. Eng.* **1**, 0068 (2017).

[16] Kalva, S. K. et al. Spiral volumetric optoacoustic tomography for imaging whole-body biodynamics in small animals. *Nat. Protoc.* **18**, 2124–2142 (2023). 37208409 10.1038/s41596-023-00834-7

[17] Choi, S. et al. Deep learning enhances multiparametric dynamic volumetric photoacoustic computed tomography in vivo (DL-PACT). *Adv. Sci.* **10**, 2202089 (2023). 10.1002/advs.202202089 PMC9811490 36354200

[18] Lin, L. et al. High-speed three-dimensional photoacoustic computed tomography for preclinical research and clinical translation. *Nat. Commun.* **12**, 882 (2021). 33563996 10.1038/s41467-021-21232-1 PMC7873071

[19] Ozcan, B. B. et al. Current status of optoacoustic breast imaging and future trends in clinical application: is it ready for prime time?. *Eur. Radiol.* **34**, 6092–6107 (2024). 38308678 10.1007/s00330-024-10600-2 PMC11297194

[20] Ku, G. et al. Multiple-bandwidth photoacoustic tomography. *Phys. Med. Biol.* **49**, 1329–1338 (2004). 15128208 10.1088/0031-9155/49/7/018

[21] Chekkoury, A., Gateau, J. & Ntziachristos, V. Multiple bandwidth volumetric optoacoustic tomography using conventional ultrasound linear arrays. Proceedings of SPIE 8800, Opto-Acoustic Methods and Applications. Munich, Germany: SPIE, 2013, 880003.

[22] Zheng, Q. C. et al. Thin ceramic PZT dual- and multi-frequency pMUT arrays for photoacoustic imaging. *Microsyst. Nanoeng.* **8**, 122 (2022). 36407887 10.1038/s41378-022-00449-0 PMC9668999

[23] Wang, H. R. et al. Development of dual-frequency PMUT arrays based on thin ceramic PZT for endoscopic photoacoustic imaging. *J. Microelectromech. Syst.* **30**, 770–782 (2021). 35528228 10.1109/jmems.2021.3096733 PMC9075345

[24] Cai, J. X. et al. Beyond fundamental resonance mode: high-order multi-band ALN PMUT for in vivo photoacoustic imaging. *Microsyst. Nanoeng.* **8**, 116 (2022). 36389053 10.1038/s41378-022-00426-7 PMC9643525

[25] Park, J. S. et al. Dual-frequency piezoelectric micromachined ultrasound transducer based on polarization switching in ferroelectric thin films. *Microsyst. Nanoeng.* **9**, 122 (2023). 37794984 10.1038/s41378-023-00595-z PMC10545730

[26] Chee, R. K. W. et al. Multifrequency interlaced CMUTs for photoacoustic imaging. *IEEE Trans. Ultrason. Ferroelectr. Freq. Control* **64**, 391–401 (2017). 28113748 10.1109/TUFFC.2016.2620381

[27] Subochev, P. V. et al. Ultrawideband high density polymer-based spherical array for real-time functional optoacoustic micro-angiography. *Light Sci. Appl.* **14**, 239 (2025). 40623976 10.1038/s41377-025-01894-y PMC12234755

[28] Shnaiderman, R. et al. A submicrometre silicon-on-insulator resonator for ultrasound detection. *Nature* **585**, 372–378 (2020). 32939068 10.1038/s41586-020-2685-y

[29] Westerveld, W. J. et al. Sensitive, small, broadband and scalable optomechanical ultrasound sensor in silicon photonics. *Nat. Photonics* **15**, 341–345 (2021).

[30] Hazan, Y. et al. Silicon-photonics acoustic detector for optoacoustic micro-tomography. *Nat. Commun.* **13**, 1488 (2022). 35304481 10.1038/s41467-022-29179-7 PMC8933411

[31] Pan, J. S. et al. Parallel interrogation of the chalcogenide-based micro-ring sensor array for photoacoustic tomography. *Nat. Commun.* **14**, 3250 (2023). 37277353 10.1038/s41467-023-39075-3 PMC10241812

[32] Shnaiderman, R. et al. Fiber interferometer for hybrid optical and optoacoustic intravital microscopy. *Optica* **4**, 1180–1187 (2017).

[33] Azhari, H. Basics of Biomedical Ultrasound for Engineers. (Hoboken: John Wiley & Sons, 2010).

[34] Hatami, M. et al. Noninvasive tracking of embryonic cardiac dynamics and development with volumetric optoacoustic spectroscopy. *Adv. Sci.* **11**, 2400089 (2024). 10.1002/advs.202400089 PMC11165471 38526147

[35] Liu, S. R. et al. Validation of photoacoustic/ultrasound dual imaging in evaluating blood oxygen saturation. *Biomed. Opt. Express* **13**, 5551–5570 (2022). 36425613 10.1364/BOE.469747 PMC9664893

[36] Seeger, M. et al. Pushing the boundaries of optoacoustic microscopy by total impulse response characterization. *Nat. Commun.* **11**, 2910 (2020). 32518250 10.1038/s41467-020-16565-2 PMC7283257

[37] Rosenthal, A., Razansky, D. & Ntziachristos, V. High-sensitivity compact ultrasonic detector based on a pi-phase-shifted fiber Bragg grating. *Opt. Lett.* **36**, 1833–1835 (2011). 21593906 10.1364/OL.36.001833

[38] Rosenthal, A., Razansky, D. & Ntziachristos, V. Wideband optical sensing using pulse interferometry. *Opt. Express* **20**, 19016–19029 (2012). 23038542 10.1364/OE.20.019016

[39] Rosenthal, A. et al. Sensitive interferometric detection of ultrasound for minimally invasive clinical imaging applications. *Laser Photonics Rev.* **8**, 450–457 (2014).

[40] Wissmeyer, G. et al. All-optical optoacoustic microscope based on wideband pulse interferometry. *Opt. Lett.* **41**, 1953–1956 (2016). 27128047 10.1364/OL.41.001953

[41] Rosenthal, A. et al. Embedded ultrasound sensor in a silicon-on-insulator photonic platform. *Appl. Phys. Lett.* **104**, 021116 (2014).

[42] La, T. A. et al. Bragg grating etalon-based optical fiber for ultrasound and optoacoustic detection. *Nat. Commun.* **15**, 7521 (2024). 39214964 10.1038/s41467-024-51497-1 PMC11364814

[43] Shnaiderman, R. et al. Silicon-photonics point sensor for high-resolution optoacoustic imaging. *Adv. Opt. Mater.* **9**, 2100256 (2021).

[44] Chen, N. B. et al. Simultaneous head-mounted imaging of neural and hemodynamic activities at high spatiotemporal resolution in freely behaving mice. *Sci. Adv.* **11**, eadu1153 (2025). 40117369 10.1126/sciadv.adu1153 PMC11927632

