# Supplementary material for: Dual-frequency fiber-array photoacoustic computed tomography for high-resolution deep brain imaging
Source: Light Sci Appl. 2026 Jun 1;15:257. doi: 10.1038/s41377-026-02324-3 (PMC13226667; doi:10.1038/s41377-026-02324-3)
Supplement: Supplementary file 1 — Supplementary material [file 41377_2026_2324_MOESM1_ESM.docx]

**Supplementary Information for**

**Dual-frequency fiber-array photoacoustic computed tomography for high-resolution deep brain imaging**

**Zitao Chen^1,2,†^, Yuhan Wu^1,2,†^, Hexiang Xu^1,2,†^, Lanling Liang^3^, Jun Ma^1,2,^*, Yi Zhang^3,^*, and Bai-Ou Guan^1,2,^***

*^1^Guangdong Provincial Key Laboratory of Optical Fiber Sensing and Communications, Institute of Photonics Technology, Jinan University, Guangzhou 510632, China;*

*^2^College of Physics & Optoelectronic Engineering, Jinan University, Guangzhou 510632, China;*

*^3^College of Life Science and Technology, Jinan University, Guangzhou 510632, China;*

*†These authors contribute equally to this work.*

********Corresponding authors*

[*jun.ma@jnu.edu.cn*](mailto:jun.ma@jnu.edu.cn)*;* [*zhangyi_0424hot@163.com*](mailto:zhangyi_0424hot@163.com)*; tguanbo@jnu.edu.cn*

**This PDF file includes:**

**Supplementary Notes 1 to 19**

**Figs. S1 to S18**

**Tables S1 to S2**

**References (1 to 22)**

**Supplementary Note 1 |** **Simulations of the frequency responses to plane ultrasound waves for fibers with and without polymer coating.**

The interaction between the optical fiber and the PA waves is modelled using the Pressure Acoustics Interface of COMSOL Multiphysics 6.0 in the frequency domain. The two-layer structure comprising the silica fiber and the surrounding water is built to model the interaction between the bare fiber and the ultrasound, and the three-layer structure with the polymer layer added as the middle layer is built for the polymer-coated fiber. Cylindrical wave radiation is applied to the outermost boundary of the structure to simulate the incident ultrasound wave on the fibers. The ultrasound impinges on the fibers and induces strains in the fibers, which is calculated using the Solid Mechanics Interface. An Acoustic-Structure Boundary multiphysics coupling is applied at the interface between the domains of the surrounding water and the fiber surface (for the bare fiber case) or polymer surface (for the coated fiber case). The strains and stresses within the fibers calculated by the above multi-physics model are coupled to the optical properties of the fibers in the form of the refractive index change, as described by the elasto-optic tensor^1^. The resulting phase shifts of the light in *x* and *y* polarizations inside the fiber are given by,

$\Delta\beta_{x}=2k_{0}n\int_{-L/2}^{L/2} \left[\varepsilon_{z}-\frac{n^{2}}{2}\left(p_{11}\varepsilon_{x}+p_{12}\left(\varepsilon_{y}+\varepsilon_{z}\right)\right)\right]dz$ (S1)

$\Delta\beta_{y}=2k_{0}n\int_{-L/2}^{L/2} \left[\varepsilon_{z}-\frac{n^{2}}{2}\left(p_{11}\varepsilon_{y}+p_{12}\left(\varepsilon_{x}+\varepsilon_{z}\right)\right)\right]dz$ (S2)

where *k*_0_ is the free-space wave number, *n* is the refractive index of the silica fiber, *L* is fiber length, *ε_x_*, *ε_y_* and *ε_z_* are the principal strains, and *p*_11_ and *p*_12_ are the Pockel’s coefficients.

According to Eqs. (S1) and (S2), the ultrasound induced shifts in the beat frequency of the orthogonally polarized light Δ*f* can be obtained by calculating the birefringence change Δ*B* based on the relationship as given in Eq. (S3).

$\Delta f=\frac{c}{n\lambda}\Delta B=\frac{c}{n\lambda}\frac{\Delta\beta_{x}-\Delta\beta_{y}}{k_{0}}$ (S3)

where *λ* is the laser wavelength and *c* is the light speed in vacuum.

The calculated ultrasound response of both the bare fiber and polymer-coated fiber, over the frequency range from 0 to 25 MHz, is plotted in Fig. 1 (see the main text). For the experiment, the variations of the optical beat frequency are read out by an I/Q demodulation unit to restore the birefringence change Δ*B* and the corresponding amplitude of the PA signals^2^.

**Fig. S1.** **Schematic of the multi-layer structures of the fibers without and with the polymer coating in the simulation model. a,** Bare fiber. Layer 1: water, Layer 2: silica fiber. **b,** Polymer-coated fiber. Layer 1: water, Layer 2: polymer, Layer 3: silica fiber.

**Supplementary Note 2 |** **Frequency responses of the FUTs with different coating lengths.**

The frequency responses of the FUTs with different polymer coating lengths are compared by mechanically scanning the PA source along the fiber axis, as shown in Fig. S2a. The PA source is prepared by uniformly covering the surface of a 250 μm optical fiber with a thin layer of carbon-black ink. The ink-covered fiber is positioned perpendicular to the FUT. The 2-D frequency responses of the FUTs with different coating lengths are shown in Fig. S2b, and their integrals along the fiber axis are shown in Fig. S2c and compared in Fig. S2d. The polymer-coated part exhibits stronger response at low frequencies compared to the bare part and the response is uniformly distributed along the fiber axis. A gradual transition of the frequency response can be observed at the interfaces of the FUTs with and without the polymer coating. As the FUT can be considered as a line-shaped detector, the output signal is the integral of the ultrasound response over the full length of the FUT sensing region. Therefore, the ratio of the low-frequency to high-frequency response can be easily tuned by adjusting the coating length to meet practical application requirements.

**Fig. S2.** **Frequency responses of the FUTs with different polymer coating lengths. a,** Schematic of the PA source scanned axially along the FUT. **b,** The frequency responses along the axes of three FUTs with different coating lengths. The length of the fully-coated region is ~ 24 mm, with the corresponding 2/3- and 1/3-coated lengths of 16 mm and 8 mm, respectively. The vertical axis indicates the frequency, and the horizontal axis denotes the position of the FUTs. **c,** The integrals of the frequency responses of the FUTs with different coating lengths along the fiber axes. **d,** Comparison of integrals in (**c**). *L_C1_*, *L_C2_*, *L_C3_*: 8 mm, 16 mm, 24 mm.

**Supplementary Note 3 |** **Parallel demodulation of FUT array elements.**

The output signals from eight channels of the FUT array during the imaging process of a hair phantom located near the array center is characterized. The FUT array is circularly scanned for 208 steps, where each channel acquires one PA signal, i.e., the A-line. After the full scanning, each channel collects 208 A-lines and the eight groups of A-lines are sequentially concatenated, as shown in Fig. S3a. The inset shows the cross-sectional image of the hair. By picking out one A-line from each of the eight groups at the same scanning step, it can be observed in Fig. S3b that the output signal from each channel exhibits similar signal amplitudes, verifying the parallel demodulation capability of the FUT array elements. The 3*σ* noise floors of the eight FUTs are shown in Fig. S3c. According to the calibrated acoustic sensitivity of the FUT^3^, which is 2.25 MHz kPa^-1^, the corresponding noise-equivalent pressure (NEP) is ~ 5.2 Pa. To verify the stability of the system during mechanical scanning, the FUT array-based PACT system is used to image a human hair phantom. Analysis of the acquired B-scan of the hair cross section (Figs. S3d,e) gives the signal amplitudes and noise levels for all the elements during the scanning. There is no significant fluctuation in the noise levels, verifying the stable imaging performance of the system during circular mechanical scanning.

**Fig. S3. The output PA signals from the FUT array-based PACT system.** **a,** B-scan acquired by the FUT array. Inset: Reconstructed cross-sectional image of the hair phantom. **b,** A-lines from each channel at the same scanning step selected from (**a**). **c,** The measured 3*σ* noise floor for each element of the FUT array. **d,** Signal-to-noise ratios (SNRs) of the PA signals for each sampling point. The purple curve shows the smoothed SNR trend. **e,** The fluctuation of the system 3*σ* noise floor during motion.

**Supplementary Note 4 | Comparison of the leaf phantom images acquired by FUT with and without focusing.**

The same leaf phantom is imaged by a curved FUT (curvature radius: 4 cm) with the sheet-like focusing and a straight FUT without focusing. The reconstructed images are compared in Figs. S4a,b, and the profiles along the white dashed lines are plotted in Fig. S4c, emphasizing the importance of the focusing capability to boost up the image contrast.

**Fig. S4. Comparison of the-leaf phantom images acquired by FUT with and without focusing. a,** Images acquired by the curved FUT with focusing. **b,** Images acquired by the straight FUT without focusing. **c,** Profiles along the white dashed lines marked in (**a**) and (**b**)**.**

**Supplementary Note 5 | The spatial responses of the FUT under different bending radii of curvature.**

**Fig. S5. The spatial responses of the FUT under different bending radii of curvature. a.** Schematic of the spatial distribution of the FUT ultrasound focus. **b.** 2-D mapping of the response amplitudes at the foci in the *x-y* plane for FUTs with different curvature radii. **c.** Profiles along the *y* axis at *x* = 0 from (**b**). **d.** 2-D mapping of the response amplitudes at the foci in the *y-z* plane for FUTs with different curvature radii.

**Supplementary Note 6 |** **Simulated spatial responses of the FUTs.**

The ultrasound spatial responses of the focused FUT are simulated using MATLAB k-wave toolbox^4,5^. The k-wave grid used for simulation is 1200 × 3600 with a single grid size of 25 μm × 25 μm (corresponding to a physical domain size of 3 cm × 9 cm). The propagation speed of ultrasound in the homogeneous medium is 1500 m s^-1^. The time-varying pressure source is defined by assigning a binary source mask with a time-varying source input, which consists of grid points occupied by two symmetrical arcs with radii of 4 cm and 3 cm. Their foci locate at the center of the k-wave grid. Each grid point is set as a point detector to record the ultrasound wave. The root-mean-square (RMS) value of the recorded signal at each grid point is extracted to form the 2-D spatial response of the focused FUT.

The two-dimensional k-wave simulations of two symmetrically arranged focused FUTs demonstrate their ultrasound field distributions in the *y-z* plane, as shown in Figs. S6a,c. As the frequency increases, the dimensions along both the *y* and *z* axes of the ultrasound focus reduce. The normalized amplitude profiles across the grid center along the *y* and *z* axes are extracted (Figs. S6b,d) and the full widths at half maximum (FWHM) of these profiles are listed in Table S1.

**Table S1 |** **FWHM values of the profiles cross the foci along the *y* and *z* axes (mm).**

| Radius (cm) | Profile direction | Frequency (MHz) | | | |
| --- | --- | --- | --- | --- | --- |
|  |  | 2.5 | 5 | 10 | 20 |
| 4 | *y* axis | 15.677 | 8.104 | 4.087 | 2.042 |
|  | *z* axis | 1.223 | 0.612 | 0.306 | 0.153 |
| 3 | *y* axis | 9.105 | 4.632 | 2.324 | 1.159 |
|  | *z* axis | 0.929 | 0.465 | 0.232 | 0.116 |

**Fig. S6.** **Simulated ultrasound fields of the focused FUTs in the *y-z* plane using MATLAB k-wave toolbox.** **a,c,** Simulated ultrasound fields in the *y-z* plane for the FUTs with radii of 4 cm and 3 cm at frequencies of 2.5 MHz and 10 MHz. The images on the right show magnified views of the focal region as denoted by the white dashed box in (**a**). **b,d,** Normalized amplitude profiles extracted across the centers along the *y* and *z* axes in (**a**) and (**c**).

**Supplementary Note 7 | Simulated field of view for the FUT array-based PACT system.**

Simulation of the field of view (FOV) on the focal plane for the FUT array-based PACT system has been performed. The simulation parameters are set as follows: the voxel size of the computational grid is 50 μm, the radius of the arc-shaped array is 4 cm, the cavity length is 22 mm, the angular interval between adjacent FUTs is 18.75°, and the coverage angle of the ring is set to 180 degrees. In the simulation, each unit length on the FUT is regarded as an independent point acoustic source emitting acoustic waves to the surroundings, and the spherical wave diffusion equation is adopted to describe the acoustic wave propagation. Meanwhile, combined with the operating characteristics of the dual-frequency FUT, the acoustic source frequencies are set to 2.5 MHz and 10 MHz, respectively. The spatial response of a single FUT can be obtained from the sound pressure received at each grid point and the sum of the spatial responses of the eight FUTs gives the spatial response of the array system. The specific simulation results are shown in Fig. S7. In general, the system FOV increases with the ultrasound wavelength. As the frequency varying from 2.5 MHz to 10 MHz, the FOV reduces from ~ 30 mm to ~ 10 mm.

**Fig. S7. Simulated spatial responses of the FUT array-based PACT system. a,b,** 2-D plot of simulated spatial response of the FUT array at the ultrasound frequency of 2.5 MHz and 10 MHz. Profiles along the dashed lines in *x* and *y* axes and the corresponding FWHM values at the ultrasound frequency of (**c, d**) 2.5 MHz and (**e, f**) 10 MHz.

**Supplementary Note 8 | Size characterization of the mouse brain vasculature.**

As shown in Fig. S8a, two blood vessels in the cerebral cortex region (R_1_) and deep brain region (R_2_) of the whole mouse brain are selected to demonstrate the imaging resolution of the FUT array-based PACT system in biological tissues. By plotting the profiles of the vessels along the white dashed lines in the regions R_1_ and R_2_ and performing the shift-and-sum process^6^, the vessel in the cerebral region has a diameter of ~ 70 μm as estimated from the FWHM of the envelope obtained from the sum of the original and delayed profiles as shown in Fig. S8b. This value is consistent with the resolution of the system evaluated by imaging the microspheres. Up to a depth of ~ 5 mm, the blood vessel with a diameter of ~ 130 μm can still be resolved.

**Fig. S8.** **Size characterization of the blood vessels in the cerebral cortex and deep brain of the mouse brain. a,** The whole mouse brain image with the selected cerebral cortex region (R_1_) and deep brain (R_2_) region marked by the solid white boxes. Diameter estimation for the selected vessels **b,** in R_1_ and **c,** in R_2_. In both (**b**) and (**c**), the green and yellow dashed curves are the profiles along the dashed line and that with a distance shift, respectively, and the purple curve is the envelope of the sum of the two dashed curves.

**Supplementary Note 9 |** **Effect of the transducer bandwidth on imaging performance.**

The effect of the transducer frequency bandwidth on the quality of the reconstructed images is simulated using MATLAB k-wave toolbox^4,5^. The number of simulation grids is 1000 × 1000 with the grid size of 25 μm × 25 μm, and thus the maximum supportable frequency is 30 MHz. Two simulation objects are set around the center of the grid including a set of general geometries and an irregular vessel phantom. The general geometries include circular phantoms with diameters of 2, 1, 0.5, and 0.25 mm and square phantoms with edge lengths of 2, 1, 0.5, and 0.25 mm. The vessel phantom has a largest vessel diameter of 500 μm and a smallest one of 50 μm. The transducer array comprises 4400 elements that evenly distribute along a circle with a radius of 12 mm in the simulation. Two Gaussian filters with the center frequencies (CFs) and bandwidths (BWs) of 10 MHz, 22 MHz and 100%, 50% are set to simulate the ultrasound response of the bare fiber. Four Gaussian filters with CFs of 2.5 MHz, 8 MHz, 15 MHz, 22 MHz and BWs of 100%, 50%, 40%, 25% are set to simulate the ultrasound response of polymer-coated FUTs. Acquisition of the ultrasound signals from the phantoms and the image reconstruction based on the time reversal algorithm are performed using the ‘kspaceFirstOrder2DG’ function in the toolbox. No additional background noise is added before the image reconstruction.

As shown in Fig. S9, the results show the initial pressure distribution of the phantoms and the reconstruction images obtained from transducer array with different frequency responses. The full-bandwidth transducer array provides the image with the highest similarity to the ground-truth phantom structure. The bare fiber transducer array retrieves the sharp boundaries of the phantoms but misses most information of the internal structures. The coated fiber transducer array with dual-frequency band can restore both the boundaries and most of the internal structures of the phantoms.

**Fig. S9.** **Reconstructed phantom images obtained by the transducer array of full-frequency band, single-frequency band and dual-frequency bands as simulated using MATLAB k-wave toolbox.**

**Supplementary Note 10 | Quantitative sO_2_ measurement of vascular phantoms in different sizes.**

Three polyethylene (PE) tubes with inner diameters (IDs) of 100 μm, 200 μm, and 300 μm, respectively, and a uniform wall thickness of 50 μm are used to simulate multi-sized blood vessels. The fixed PE tubes are embedded in a mixture of 3% agar and 1% intralipid emulsion. The PE tubes are connected end to end, parallel to each other and placed ~ 1 mm apart, and the blood inside always comes from the same source. The FUT is placed in the same plane as the vascular phantom, and the focal plane is orthogonal to the vessels. Pulsed laser excitation at 850 nm and 750 nm with a spot diameter of ~ 5 mm is delivered to the vascular phantom through the optical fiber bundles. Each oxygen saturation (sO_2_) collection session consists of 30 s for data acquisition under the laser illumination at two different wavelengths. Fresh anticoagulated bovine blood stored at 4 ℃ is injected into the tubes (pre-filled with blood) at a rate of 0.5 μL min^-1^ using a syringe pump (LSP02-1B, LongerPump). The blood sample is replaced each time once a sO_2_ measurement has completed.

The six parallel results obtained from the same experiment are shown in Fig. S10. The original sO_2_ maps and statistical results reveal highly consistent sO_2_ values for the three different sized vessels, which confirms the ability of FUT to restore PA signals and sO_2_ information for targets of different sizes. Based on the sO_2_ maps obtained after low-pass (LP) filtering and high-pass (HP) filtering (cut-off frequency: 5 MHz), the low-frequency response of the FUT plays an important role in accurate and stable sO_2_ quantification for vessels with IDs of 200 μm and 300 μm. It can be observed that the sO_2_ maps after HP filtering only show the edges of the vessel, rather than the inside blood, which provides more representative sO_2_ information. The situation differs as the size of the blood vessel is further reduced to 100 μm. The low-frequency PA signals of the 100 μm vessel reduces in the amplitude, resulting in the loss of information in sO_2_ mapping after LP filtering and thus degradation in the accuracy of sO_2_ quantification. Interestingly, by combining both the low- and high-frequency responses, FUT can accurately reproduce the sO_2_ levels of blood vessels at 100 μm compared with the results based on single low- or high-frequency response. As a result, the dual-frequency response of FUT can benefit the high-accuracy sO_2_ quantification for vessels with different sizes, ranging from tens of micrometers to hundreds of micrometers, which is important for sO_2_ quantification considering the varying feature sizes of biological vasculatures.

**Fig. S10. Quantitative sO_2_ measurement of vascular phantoms with different sizes.** **a,** Schematic of the experimental setup for quantitative sO_2_ mapping of the blood-filled tube phantom embedded in intralipid-mixed agar. **b,** Photographs of the blood-filled tubes. **c**, PA images of the blood-filled tubes before and after masking. **d,** Six sets of parallel experimental sO_2_ maps with no filtering, LP and HP filtering. **e,** Statistical analysis of sO_2_ values of the blood-filled tubes with three different sizes. The values in the same subgroup in **d**, from left to right, represent results 1 to 6, respectively. Bars and error bars represent normalized sO_2_ means and standard deviation (SD).

**Supplementary Note 11 | Quantitative measurement of sO_2_ at different flow velocities.**

**Fig. S11. Quantitative sO_2_ measurement of the vascular phantom at different flow velocities. a,** Schematic of the experimental setup for quantitative sO_2_ mapping of the blood-filled tube phantom embedded in intralipid-mixed agar. A syringe pump is used to control the flow velocities^7,8^. For the tube with an ID of 300 μm, by setting the pump flow rate to 0, 21, 42, 63, and 84 μL min^-1^, the flow velocities are approximately 0, 5, 10, 15, and 20 mm s^-1^, respectively^9,10^. **b,** PA images of the blood-filled tubes before and after masking. **c,** Experimental sO_2_ maps of the vascular phantom at five flow velocities with no filtering, LP and HP filtering. **d,** Statistical analysis of sO_2_ values from static blood before and after filtering. The values in the same subgroup represent the three vessels, respectively. Numbers 1 to 4 correspond to the four subgroups. Bars and error bars represent normalized sO_2_ means and SD. **e,** Statistical analysis of sO_2_ values at five flow velocities without filtering. **f,** Mean sO_2_ values within the green dashed box in (**e**). **g,** Statistical analysis of sO_2_ values at five flow velocities under LP filtering. **h,** The mean sO_2_ values within the green dashed box in (**g**).

**Supplementary Note 12 |** **Comparison of the mouse brain sO_2_ maps derived from different frequency components of the PA signals*.***

A comparative analysis of low- and high-frequency sO_2_ maps in the mouse brain obtained at 20% O_2_ concentration is performed. The PA signals excited by the 750 nm and 850 nm light are processed with the 3^rd^ LP and HP Butterworth filter (cut-off frequency: 3 MHz) to separate the low- and high-frequency components.

**Fig. S12.** **sO_2_ maps obtained from original (OG), LP-filtered (LP) and HP-filtered (HP) PA signals. a,** Mouse cerebral cortex and **b,** brain coronal section showing original, LP-filtered, and HP-filtered sO_2_ maps. **c,** Statistical distributions of sO_2_ in cortical vessels V_1_-V_4_. **d,** Coronal regions R_1_-R_4_. The horizontal lines denote the median values.

Figs. S12a,b present the corresponding low- and high-frequency sO_2_ maps of the mouse cerebral cortex and coronal section via dual-wavelength sO_2_ calculation. The sO_2_ levels in four major cortical vessels (V_1_-V_4_) and four distinct regions in the coronal section (R_1_-R_4_) are quantitatively analyzed and plotted in Figs. S12c,d. The superior sagittal sinus (SSS) with large feature size as denoted as V_1_, shows the least fluctuation in the restored sO_2_ results. The high consistency between the original and LP-filtered sO_2_ measurements clearly reflects the sO_2_ level of this vessel. V_2_ and V_3_, being the branch vessels, exhibit similar sO_2_ results to V_1_, although the LP-filtered values show slightly more fluctuations due to the smaller sizes. Statistically, the finer vessels denoted as V_4_ show larger fluctuations. The coronal sO_2_ maps are segmented into shallow layers R_1_, R_2_ and deep layers R_3_, R_4_ for quantitative analysis. R_1_ and R_2_ have fine cortical blood vessels, exhibiting larger fluctuations in both original and HP-filtered sO_2_ maps and statistical results. R_3_ and R_4_ are vascular structures of the rostral rhinal vein (RRV) and anterior choroidal artery (AchA) featuring large sizes, showing good consistency in sO_2_ maps and statistical results.

**Supplementary Note 13 |** **sO_2_ mapping in oxygen challenge.**

In the O_2_ challenge test, the mouse inhales a mixture of anesthesia and O_2_ continuously and the O_2_ concentration is altered to 100% for ~ 80 s prior to the test. The following O_2_ challenge process includes three phases: continuous intake of 100% O_2_ for 62.4 s, 10% O_2_ for 124.8 s, and 100% O_2_ for 62.4 s. The detailed experimental flowchart is illustrated in Fig. S13a.

**Fig. S13.** **Rapid mouse** **cerebral sO_2_ mapping during the O_2_** **challenge obtained by down-sampling the PA dataset. a,** Flowchart of the mouse cerebral sO_2_ mapping obtained during the O_2_ challenge for O_2_ altered from 100% to 10% and then to 100%. **b,** PA images of the mouse brain coronal section reflecting the temporal changes in sO_2_. **c,** Coronal section PA image of the mouse brain, showing the four selected vascular regions (R_1_–R_4_) for sO_2_ monitoring. **d,** Temporal dynamics of sO_2_ in the four vascular regions (R_1_–R_4_). Each data point represents the average sO_2_ value within the corresponding vascular region.

Multispectral PACT is performed continuously with dynamic switching of the inhaled O_2_ concentrations (100% O_2_ → 10% O_2_ → 100% O_2_) following the above durations. The excitation wavelengths for sO_2_ mapping are switched between 850 nm and 750 nm, and the imaging duration for each wavelength is 20.8 s. The sO_2_ map is obtained in sequence as follows: the sO_2_ map 1 is calculated using the paired 850 nm and 750 nm imaging results, the sO_2_ map 2 is calculated using the previous 750 nm and the newly acquired 850 nm imaging results, the sO_2_ map 3 is calculated using the previous 850 nm and the newly acquired 750 nm imaging results, in a sequential sliding manner. The process is repeated until all sO_2_ maps are obtained. To explore the potential for rapid sO_2_ mapping, the obtained PA dataset is down-sampled for 10 times to simulate sparse scanning of the FUT array, as shown in Fig. S13b. The dynamic changes in sO_2_ during the O_2_ challenge can be still visualized and agree well with the O_2_ supply.

**Supplementary Note 14 | Comparison of the GBM images by MRI and PACT.**

MRI and PACT are both performed on the glioblastoma (GBM)-bearing mouse model. Seven cross-sections are acquired from +0.5 mm to -2.5 mm relative to bregma, at 0.5 mm intervals, as shown in Fig. S14. The contours of the abnormal tissue can be clearly observed at different locations in the PA images, which match well with the MRI images. The mouse is further skull thinned to reduce the ultrasound attenuation and distortion, which makes the tumor contours clearer and several vessels inside the tumor visible, compared with the noninvasive PA images. We delineate the boundary of the tumor region in the MRI images using ITK-SNAP, a standard segmentation and registration toolkit Firstly, the region of interest (ROI) encompassing the tumor is selected and automatic contrast adjusting is performed. Then, the polygon tool is used to manually select and connect the points at the tumor boundary. Finally, the obtained tumor contours are used as the segmentation reference and directly applied to the corresponding regions of the presented PA images to confirm the observed tumor vasculature as acquired. We currently do not perform direct segmentation on the PA images due to the high heterogeneity and ill-defined margins of the tumor vasculature. Direct segmentation might be attempted by combining the machine learning model after collecting more PA images of the brain tumor vasculature and building the database^11,12^.

**Fig. S14. Cerebral coronal section images of the GBM-bearing mouse model as acquired via MRI and PACT.** Scale bar: 2 mm.

**Supplementary Note 15 |** **Pathologic analysis of the GBM.**

Fig. S15 shows the hematoxylin and eosin (H&E) staining images of the mouse brain paraffin sectioning at post-inoculation day (PID) 18 after GBM inoculation. In the GBM region, hypercellularity with atypical cellular morphology and enlarged nuclei can be observed, compared with the surrounding normal brain tissues, which confirms the success of the GBM inoculation. The dark hyperchromatic nuclei are attributed to the high DNA content, which is indicative of rapid tumor cell proliferation, during which tumor cells require sufficient nutrients and O_2_ to support their growth^13^. This result explains the relatively high sO_2_ in the tumor-grown area as observed from the PA images as shown in Fig. 5 in the main text.

**Fig. S15.** **H&E-stained paraffin section of the mouse brain at PID 18 after GBM inoculation.**

**Supplementary Note 16 | Comparison of the FUT with the state-of-the-art Bragg grating-based ultrasound sensors.**

Performance comparison with the state-of-the-art Bragg grating-based optical transducers in terms of the bandwidth, sensitivity, and focus or not is given in Table S2.

**Table S2 | Comparison of the FUT with the Bragg grating-based ultrasound sensors.**

| Sensor type | Sensitivity | Bandwidth | Focused or not |
| --- | --- | --- | --- |
| π-phase-shifted fiber Bragg grating (πFBG) ^[14-18]^ | 9 - 140 mPa Hz^-1/2^ | 10 - 80 MHz | Non-focused  /Focused |
| π-phase-shifted waveguide Bragg grating (πWBG) ^[19]^ | NA | 20 MHz | Non-focused |
| Silicon waveguide–etalon detector (SWED) ^[20]^ | 9 mPa Hz^-1/2^ | 230 MHz | Non-focused |
| π-phase-shifted Bragg grating (πBG) ^[21]^ | 9.8 mPa Hz^-1/2^ | > 200 MHz | Non-focused |
| Bragg grating embedded etalon resonator (EER) ^[22]^ | 2.5 mPa Hz^-1/2^ | 160 MHz | Non-focused |
| Fiber ultrasound transducer (FUT) (this work) | 1 mPa Hz^-1/2^ | 21 MHz | Focused  (Lens-free) |

**Supplementary Note 17 | Spatial response characterization of the FUT array-based PACT system.**

To characterize the sheet-like spatial response, a point-like ultrasound source made of a carbon-black coated fiber tip illuminating by 532 nm laser pulses is mechanically scanned with a step size of 400 μm. The measured spatial responses of the single FUT and the FUT array-based PACT system are shown in Fig. S16.

**Fig. S16. Spatial response characterization of the FUT array-based PACT system. a,** Schematic of the spatial distribution of the FUT ultrasound focus. **b,** 2-D plot of the measured spatial distribution in the *x-y* plane for the single element and the full array. **c,** Profiles along the dashed lines (*x* and *y* axes) in (b) and the corresponding FWHM values.

**Supplementary Note 18 |** **Imaging resolution of the FUT array-based PACT system.**

The spatial resolution of the FUT array-based PACT system is studied by imaging a polystyrene dyed microspheres phantom which is prepared by immobilizing the 10 μm microspheres into 2% solidified agar. The phantom is placed at the rotation center of the FUT and excited by the 532 nm pulsed laser through the fiber bundles with an optical fluence of ~ 10 mJ cm^-2^. The FUT rotates 1000 steps for 180° and acquires 1000 A-lines in total for the image reconstruction. The bipolar microsphere image is shown in Fig. S17a. The resolution of the FUT array-based PACT system is determined using the shift-and-sum process^6^ similar to that as described in Supplementary Note 8. The system exhibits an axial resolution of ~ 68 μm and a lateral resolution of ~ 74 μm, as shown in Figs. S17b,c.

**Fig. S17.** **Resolution of the FUT array-based PACT system characterized by imaging the polystyrene-dyed** **microsphere.** **a,** The bipolar image of the microsphere. **b,c,** The green and yellow dashed curves are the profiles along the dashed lines in (**a**) and that with a distance shift, respectively. The purple curve is the envelope of the sum of two dashed curves.

**Supplementary Note** **19 |** **Setup of eight-channel FUT array-based PACT system.**

The optical parametric oscillator (OPO) laser (Radiant QX8120, Opotek) outputs pulsed light at the desired wavelength for imaging with a repetition frequency of 10 Hz and a pulse width of 6.5 ns. The pulsed light transmits through the fiber bundles (consisting of 7200 fibers, 6 mm aperture, Y-shaped) to the imaging objects to excite the PA signals, as shown in Fig. S18. For each channel, the 980 nm pump light (HYLM-980-CW, Tianjin UniStarCom Technology) with a power of ~ 250 mW is fed into the FUT through a wavelength division multiplexer (WDM). The 1550 nm laser light output from the FUT is modulated by ultrasound signals. After traveling through the WDM, polarization controller, isolator and polarizer, the ultrasound-modulated light is received by a photodetector (PD, KG-PD-8-10G-A-SM-FC, Conquer). Due to the intrinsic fiber birefringence, the output light includes two orthogonal polarization modes that beat with each other and generate a radio frequency (RF) signal. The ultrasound-induced stress in the fiber shifts the frequency of the RF signal, which is subsequently down-converted by a frequency mixer from GHz to MHz and digitized by a DAQ (PXIe5641, ART Technology) for I/Q demodulation^2^. Motor motion, data acquisition and OPO laser firing are controlled by the synchronous clock signals from a controller.

**Fig. S18.** **Schematic of the FUT array-based PACT system.** WDM: wavelength division multiplexer, DAQ: data acquisition unit, PC: personal computer, OPO laser: optical parametric oscillator laser.

**Supplementary References**

1. Flax, L. et al. Acoustically induced birefringence in optical fibers. *J. Opt. Soc. Am.* **72**, 1159-1162 (1982).

2. Xu, H. et al. B. O. Noninvasive high-resolution deep-brain photoacoustic imaging with a negatively focused fiber-laser ultrasound transducer. *Photonics Res.* **12**, 2996 (2024).

3. Bai, X. et al. Focus-tunable fiber-laser ultrasound sensor for high-resolution linear-scanning photoacoustic computed tomography. *Appl. Phys. Lett.* **116**, 153701 (2020).

4. Treeby, B. E. & Cox, B. T. k-Wave: MATLAB toolbox for the simulation and reconstruction of photoacoustic wave fields. *J. Biomed. Opt.* **15**, 021314 (2010).

5. Treeby, B. E. et al. Rapid calculation of acoustic fields from arbitrary continuous-wave sources. *J. Acoust. Soc. Am.* **143**, 529–537 (2018).

6. Ni, L. et al. 3D imaging of aqueous veins and surrounding sclera using a dual-wavelength photoacoustic microscopy. *Biomed. Opt. Express* **14**, 6291-6300 (2023).

7. Ning, B. *et al.* Simultaneous photoacoustic microscopy of microvascular anatomy, oxygen saturation, and blood flow. *Opt. Lett.* **40**, 910 (2015).

8. Liu, C., Liang, Y. & Wang, L. Single-shot photoacoustic microscopy of hemoglobin concentration, oxygen saturation, and blood flow in sub-microseconds. *Photoacoustics* **17**, 100156 (2020).

9. Yao, J. *et al*. *In vivo* photoacoustic imaging of transverse blood flow using Doppler broadening of bandwidth. *Opt. Lett.* **35**, 1419-1421 (2010).

10. Unekawa, M. *et al.* RBC velocities in single capillaries of mouse and rat brains are the same, despite 10-fold difference in body size. *Brain Res.* **1320**, 69–73 (2010).

11. Yoon, C. *et al.* Deep learning-based virtual staining, segmentation, and classification in label-free photoacoustic histology of human specimens. *Light Sci. Appl.* **13**, 226 (2024).

12. Tong, X. *et al.* Panoramic photoacoustic computed tomography with learning-based classification enhances breast lesion characterization. *Nat. Biomed. Eng.* **10**, 161–177 (2025).

13. Balasundaram G. et al. Noninvasive anatomical and functional imaging of orthotopic glioblastoma development and therapy using multispectral optoacoustic tomography. *Transl. Oncol.* **11**,1251-1258 (2018).

14. Rosenthal, A., Razansky, D. & Ntziachristos, V. High-sensitivity compact ultrasonic detector based on a pi-phase-shifted fiber Bragg grating. *Opt. Lett.* **36**, 1833 (2011).

15. Rosenthal, A., Razansky, D. & Ntziachristos, V. Wideband optical sensing using pulse interferometry. *Opt. Express* **20**, 19016 (2012).

16. Rosenthal, A. *et al.* Sensitive interferometric detection of ultrasound for minimally invasive clinical imaging applications. *Laser Photonics Rev.* **8**, 450–457 (2014).

17. Wissmeyer, G., Soliman, D., Shnaiderman, R., Rosenthal, A. & Ntziachristos, V. All-optical optoacoustic microscope based on wideband pulse interferometry. *Opt. Lett.* **41**, 1953 (2016).

18. Shnaiderman, R. *et al.* Fiber interferometer for hybrid optical and optoacoustic intravital microscopy. *Optica* **4**, 1180 (2017).

19. Rosenthal, A. *et al.* Embedded ultrasound sensor in a silicon-on-insulator photonic platform. *Appl. Phys. Lett.* **104**, 021116 (2014).

20. Shnaiderman, R. *et al.* A submicrometre silicon-on-insulator resonator for ultrasound detection. *Nature* **585**, 372–378 (2020).

21. Hazan, Y., Levi, A., Nagli, M. & Rosenthal, A. Silicon-photonics acoustic detector for optoacoustic micro-tomography. *Nat. Commun.* **13**, 1488 (2022).

22. La, T. A., Ülgen, O., Shnaiderman, R. & Ntziachristos, V. Bragg grating etalon-based optical fiber for ultrasound and optoacoustic detection. *Nat. Commun.* **15**, 7521 (2024).
